# Forebrain-specific, conditional silencing of Staufen2 alters synaptic plasticity, learning, and memory in rats

**DOI:** 10.1186/s13059-017-1350-8

**Published:** 2017-11-17

**Authors:** Stefan M. Berger, Iván Fernández-Lamo, Kai Schönig, Sandra M. Fernández Moya, Janina Ehses, Rico Schieweck, Stefano Clementi, Thomas Enkel, Sascha Grothe, Oliver von Bohlen und Halbach, Inmaculada Segura, José María Delgado-García, Agnès Gruart, Michael A. Kiebler, Dusan Bartsch

**Affiliations:** 1 Department of Molecular Biology, CIMH and Medical Faculty Mannheim, Heidelberg University, 68159 Mannheim, Germany; 2 Division of Neurosciences, Pablo de Olavide University, 41013 Seville, Spain; 3 BioMedical Center, Medical Faculty, Ludwig Maximilians University, 82152 Planegg-Martinsried, Germany; 4 Institute for Anatomy and Cell Biology, University Medicine Greifswald, 17487 Greifswald, Germany; 5 Present Address: Institute Cajal (CSIC), 28002 Madrid, Spain

**Keywords:** RNA-binding protein, Staufen2, Conditional inducible rat knockdown, LTP, LTD, BCM rule, Working memory, Operant learning, Cognitive flexibility

## Abstract

**Background:**

Dendritic messenger RNA (mRNA) localization and subsequent local translation in dendrites critically contributes to synaptic plasticity and learning and memory. Little is known, however, about the contribution of RNA-binding proteins (RBPs) to these processes in vivo.

**Results:**

To delineate the role of the double-stranded RBP Staufen2 (Stau2), we generate a transgenic rat model, in which Stau2 expression is conditionally silenced by Cre-inducible expression of a microRNA (miRNA) targeting Stau2 mRNA in adult forebrain neurons. Known physiological mRNA targets for Stau2, such as *RhoA*, *Complexin 1*, and *Rgs4* mRNAs, are found to be dysregulated in brains of Stau2-deficient rats. In vivo electrophysiological recordings reveal synaptic strengthening upon stimulation, showing a shift in the frequency-response function of hippocampal synaptic plasticity to favor long-term potentiation and impair long-term depression in Stau2-deficient rats. These observations are accompanied by deficits in hippocampal spatial working memory, spatial novelty detection, and in tasks investigating associative learning and memory.

**Conclusions:**

Together, these experiments reveal a critical contribution of Stau2 to various forms of synaptic plasticity including spatial working memory and cognitive management of new environmental information. These findings might contribute to the development of treatments for conditions associated with learning and memory deficits.

**Electronic supplementary material:**

The online version of this article (doi:10.1186/s13059-017-1350-8) contains supplementary material, which is available to authorized users.

## Background

Targeting of messenger RNAs (mRNAs) to synapses [1] and the subsequent regulation of local synaptic translation [2] are essential for hippocampal synaptic plasticity and for learning and memory [3, 4]. Little is known about the contribution of RNA-binding proteins (RBPs) involved in RNA transport and synaptic protein synthesis in vivo. In *Drosophila*, the RBP staufen is involved in mRNA localization during oogenesis and the early development of the nervous system [5]. Unbiased genetic screens in *Drosophila* identified staufen and pumilio genes as critical players in long-term memory formation [6]. Two different genes encode for mammalian homologs of *Drosophila* staufen: the ubiquitous Staufen1 (Stau1) and the more brain specific Staufen2 (Stau2) [7, 8]. Both proteins have been implicated in dendritic mRNA transport [7, 9–11]. Stau2 knockdown reduces the number of mature dendritic spines, PSD95-positive synapses and miniature excitatory postsynaptic currents (mEPSCs) in cultured hippocampal neurons [12] and impairs chemically induced mGluR-dependent long-term depression (LTD) in organotypic, hippocampal slice cultures [13]. We have recently identified the physiological mRNA targets and the protein interactors for Stau2-containing granules [14, 15], revealing numerous targets involved in synaptic plasticity. The relevance of Stau2 in animal behavior, however, has not been tested due to lack of suitable animal models.

Here, we report a novel transgenic rat model allowing tissue-specific Stau2 silencing by tamoxifen (Tx)-inducible Cre-mediated expression of a synthetic microRNA (miRNA) selectively targeting *Stau2* mRNA. The rat was our preferred animal model as its anatomy facilitates simultaneous multi-electrode recordings from large neuronal populations in vivo [16] and they have advanced cognitive capabilities [17]. This new animal model enabled us to delineate the physiological role of Stau2 in the brain as Stau2 silencing yielded reduced dendritic spine density, enhanced long-term potentiation (LTP), impaired LTD, and altered spatial working and associative memory. Thus, Stau2, which is implicated in dendritic mRNA transport, critically contributes to synaptic plasticity and cognitive performance.

## Results and discussion

### Conditional rat model for forebrain-specific Stau2 downregulation

**Fig. 1:**
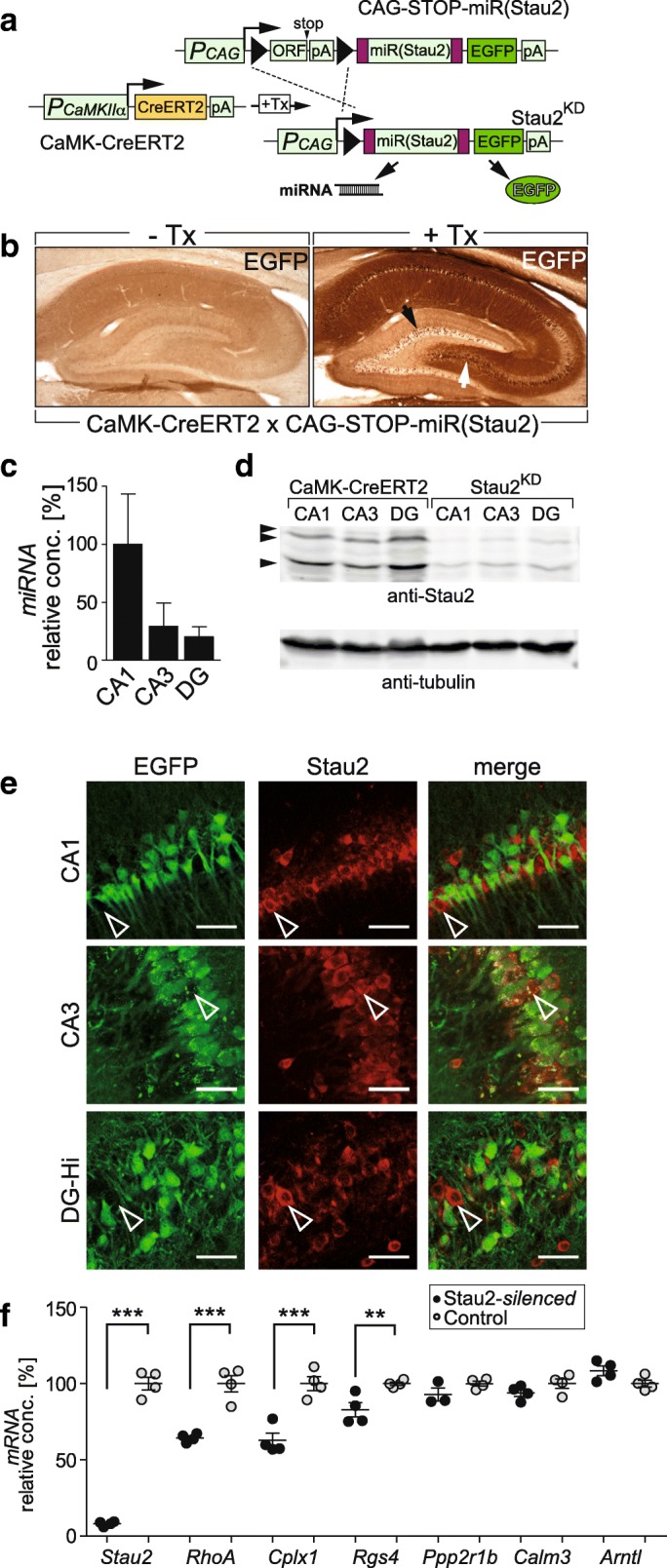
Conditional forebrain-specific Stau2 knockdown rat model. **a** *Schematic diagram* of rat transgenes used to achieve tamoxifen (Tx)-inducible, forebrain-specific Stau2 knockdown. Upon Tx application (+Tx), the inducible form of Cre recombinase (CreERT2), produced under the control of the CaMKIIα promoter (P_CaMKIIα_) deletes a *loxP*-flanked “ORF-STOP cassette” (consisting of an ORF and a polyA site (pA)). This allows simultaneous expression of the synthetic miRNA targeting Stau2 (miR(Stau2)) (included within an intronic sequence) and EGFP, driven by the CAG promoter (P_CAG_). **b** Immunohistochemical analysis of CreERT2-inducible EGFP production in the hippocampus of double transgenic CaMKIIα-CreERT2 x CAG-STOP-miR(Stau2) animals 6–8 weeks after vehicle (-Tx) or Tx (+Tx; Stau2^KD^) injection. *Arrows* indicate the hilus (*white*) or the granular cell layer (*black*) of the dentate gyrus (DG). **c** Quantification of miR(Stau2) expression by quantitative reverse transcription polymerase chain reaction (qRT-PCR) in hippocampal areas of Stau2^KD^ animals (n = 4 animals). *Bars* represent mean + SEM. **d** Representative *western blot analysis* of Stau2 protein levels in hippocampal areas of Tx-injected Stau2^KD^ and CaMKIIα-CreERT2 rats (n = 3 animals/group). *Arrowheads* indicate the different isoforms of Stau2 protein [7]. **e** Dual immunofluorescence of EGFP (Alexa488; *green*), indicating miR(Stau2) expression, and Stau2 (Cy5; *red*) in hippocampal areas CA1, CA3, and the hilus of the DG (DG-Hi) of Stau2^KD^ animals; *arrowheads* indicate Stau2-positive immunostaining in EGFP-negative neurons. Scale bars: 50 μm. **f** Quantification of mRNA levels by qRT-PCR for the indicated genes in control and ubiquitously germline Cre-recombined CAG-STOP-miR(Stau2) rats (Stau2-silenced). Each *dot* represents the corresponding mRNA level for one animal. Figure shows single values, mean ± SEM. *Stau2:* Staufen2, *RhoA:* Ras homolog family member A, *Cplx1:* Complexin 1, *Rgs4:* Regulator of small G protein signaling, *Ppp2r1b:* Protein phosphatase 2 scaffold subunit A, isoform α, *Calm3:* Calmodulin 3, *Arntl:* Aryl hydrocarbon receptor nuclear translocator like. *PPIA* (Peptidylprolyl isomerase A) was used as housekeeping gene (F_genotype*genes_ (6,41) = 47.21; *p* < 0.0001). *Stars* represent *p* values between genotypes obtained by Bonferroni post hoc analysis following two-way ANOVA of repeated measures, ** *p* < 0.01, ***p < 0.001

To construct an effective miRNA specifically targeting rat *Stau2* mRNA (miR(Stau2); Fig. 1a), we integrated the sequence of our well characterized shStau2 [12, 15] into the miRNA3-backbone [18]. The obtained miR(Stau2) was then placed into an artificial intron preceding the coding sequence of the enhanced green fluorescent protein (EGFP), enabling to monitor miRNA expression in target cells (hence Stau2 silencing) by concomitant EGFP production ([18, 19]; Additional file 1: Figure S1A). Upstream of the miR(Stau2)-EGFP transgene, we included a *floxed* “ORF-STOP cassette” preceded by the ubiquitous CAG promoter (chicken β-actin promoter together with a CMV enhancer). Microinjection of the conditional expression construct into fertilized rat oocytes yielded six transgenic CAG-STOP-miR(Stau2) founder animals.

To enable Tx-inducible recombination specific in forebrain pyramidal neurons, CAG-STOP-miR(Stau2) founders were crossed with CaMKIIα-CreERT2 rats [20] (Fig. 1a). Double transgenic littermates were injected with either vehicle (-Tx) or Tx (+Tx). In uninduced animals (-Tx), transcription from the ubiquitous CAG promoter terminated within the “ORF-STOP cassette.” In contrast, Tx-induced Cre-mediated recombination removed the “ORF-STOP cassette” enabling the co-expression of miR(Stau2) and EGFP (Fig. 1a, b). Immunostaining for EGFP on sagittal brain sections revealed that Stau2^KD^ animals derived from the founder line #17 displayed the most robust EGFP expression in the hippocampus (Fig. 1b), prefrontal cortex, and other cortical areas (Additional file 1: Figure S1B, C), compared to the other lines. Quantitative polymerase chain reaction from genomic DNA (qPCR) [21] revealed that line #17 contained 2.75 ± 0.15 integrated copies of the CAG-STOP-miR(Stau2) transgene. This line was selected for subsequent studies. Tx-treated double transgenic CAG-STOP-miR(Stau2) × CaMKIIα-CreERT2 rats were termed Stau2^KD^. Quantitative reverse transcription PCR (qRT-PCR) from forebrain extracts showed that *EGFP* mRNA in -Tx animals was ~ 5% of the levels in recombined animals (*EGFP* mRNA copies relative to *PPIA* mRNA: 0.013 ± 0.002 for -Tx animals (n = 3) and 0.254 ± 0.035 for + Tx (n = 4); *p* < 0.001 obtained by t-test), indicating low leakage of expression under basal conditions. Dual immunofluorescence for EGFP and the neuron-specific marker NeuN on brain sections from Stau2^KD^ animals revealed that the majority of neurons within prefrontal cortex (61.7 ± 1.6%; n = 3) and the rest of the neocortex (59.2 ± 7.2%; n = 3) expressed EGFP. Within the hippocampus, EGFP-positive neurons were abundant in CA1 (54.2 ± 11.7%; n = 3), CA3 (47.6 ± 9.8%; n = 3) and in the hilus of the dentate gyrus (DG) (75.1 ± 8.6%; n = 3), but only sparse in the granule cell layer of the DG (9.4 ± 1.7%; n = 3).

Transgenic expression and Stau2 silencing among hippocampal areas in Stau2^KD^ rats in vivo were analyzed by qRT-PCR, corroborating simultaneous expression of miR(Stau2) and *EGFP*, and concomitant *Stau2* depletion without *Stau1* compensatory upregulation (Fig. 1c; Additional file 1: Figure S1D–F). EGFP and Stau2 protein expression levels were evaluated within various hippocampal areas by either western blot or immunostaining, confirming EGFP expression and strong reduction of Stau2 protein concentration in Stau2^KD^hippocampal areas when compared to respective control animals (Fig. 1b, d, e; Additional file 1: Figure S1G, H). Dual immunofluorescence of Stau2 and EGFP in Stau2^KD^ brains further demonstrated that Stau2 expression was strongly reduced in EGFP-positive neurons in comparison to adjacent EGFP-negative cells both in hippocampus and cerebral cortex (Fig. 1e; Additional file 1: Figure S1H). Taken together, we established Stau2^KD^ rats as a novel valid animal model to study the contribution of Stau2 protein in adult CaMKIIα-expressing neurons, i.e. excitatory pyramidal neurons, to hippocampal synaptic plasticity and related behavior.

### Validation of physiological Stau2 functions in vivo

Stau2 silencing in rat cortical neurons in culture, using the same Stau2 target sequence, altered the expression levels of several target mRNAs [15]. Among them, *Rgs4* or *Cplx1* had previously been identified as Stau2 mRNA targets [15]. To characterize the effect of constitutive Stau2 depletion in vivo, we analyzed the mRNAs of known Stau2 targets in control and Stau2-silenced rats (constitutively recombined rat line, expressing miR(Stau2) in all the cells). Stau2-silenced rats were obtained by breeding CAG-STOP-miR(Stau2) animals with a transgenic line that constitutively expresses Cre-recombinase under the control of the EF1α promoter, targeted to the rat ROSA26 locus (see “Methods”). qRT-PCR analysis confirmed that *RhoA*, *Cplx1*, and *Rgs4* mRNAs were reduced in the forebrain of Stau2-silenced rats compared to control animals (Fig. 1f). No differences were observed for total levels of *Calm3*, *Ppp2r1b* (two known Stau2 targets) [15, 22], or *Arntl* (a known non-target of Stau2) mRNAs (Fig. 1f). However, the intron retained isoform of *Calm3* [22] was more restricted to the soma of pyramidal neurons in the cortex (specially in layer V) and the hippocampus and less present in the dendritic field of the CA1 area in Stau2-silenced animals when compared to control littermates (Additional file 1: Figure S1I), although neither the *Calm3* intron isoform nor the total *Calm3* mRNA levels were affected (Additional file 1: Figure S1J).

In cultured rat hippocampal neurons, depletion of Stau2 by RNAi lead to significant reduction in both number and size of dendritic spines [12]. To examine the effect of Stau2 downregulation on dendritic spines in vivo, we performed Golgi-Cox staining on Tx-injected control (CaMKIIα-CreERT2) and Stau2^KD^ brain slices. Analysis of the CA1 area demonstrated that dendritic spine density and length were significantly lower on apical, but not on basal dendrites (Additional file 1: Figure S1K–N). On the other hand, spine density and length of apical CA3 dendrites were not significantly altered (Additional file 1: Figure S1O, P). This difference in dendritic spines between apical CA1 and CA3 regions could be due to lower expression of the transgene (hence resulting in a higher Stau2 concentration) in the CA3 region of Stau2^KD^ animals (Fig. 1c, Additional file 1: S1G). Moreover, immunoreactivity for synapsin1 was reduced in CA1, CA3, and the hilus in Stau2-silenced rats when compared to control littermates (data not shown). Together, Stau2 in vivo depletion exerts molecular and morphological deficiencies in the hippocampus that recapitulate previous in vitro observations [12, 15, 22].

### Stau2 silencing favored enhanced synaptic strength

Regulation of local mRNA translation near synapses is linked to the modulation of long-term synaptic plasticity [1]. We therefore investigated the impact of Stau2 knockdown on hippocampal synaptic plasticity in vivo. Tx-treated Stau2^KD^, CaMKIIα-CreERT2, and CAG-STOP-miR(Stau2) rats were implanted with stimulating electrodes in the dorsomedial part of the right angular bundle to activate medial perforant pathway (PP) synapses and with recording electrodes in CA1 and CA3 areas. Determination of the input/output relationship by presenting single pulses of increasing intensity to the ipsilateral PP resulted in comparable increases in evoked field excitatory postsynaptic potential (fEPSP) slopes in all recorded areas in all three groups of rats (Additional file 1: Figure S2A, B). Animals also displayed similar paired pulse facilitation by showing a similar response in the facilitation evoked by the second pulse at different interstimulus intervals (Additional file 1: Figure S2C, D). These experiments suggested that Stau2 did not influence short-term plasticity in forebrain neurons.

**Fig. 2:**
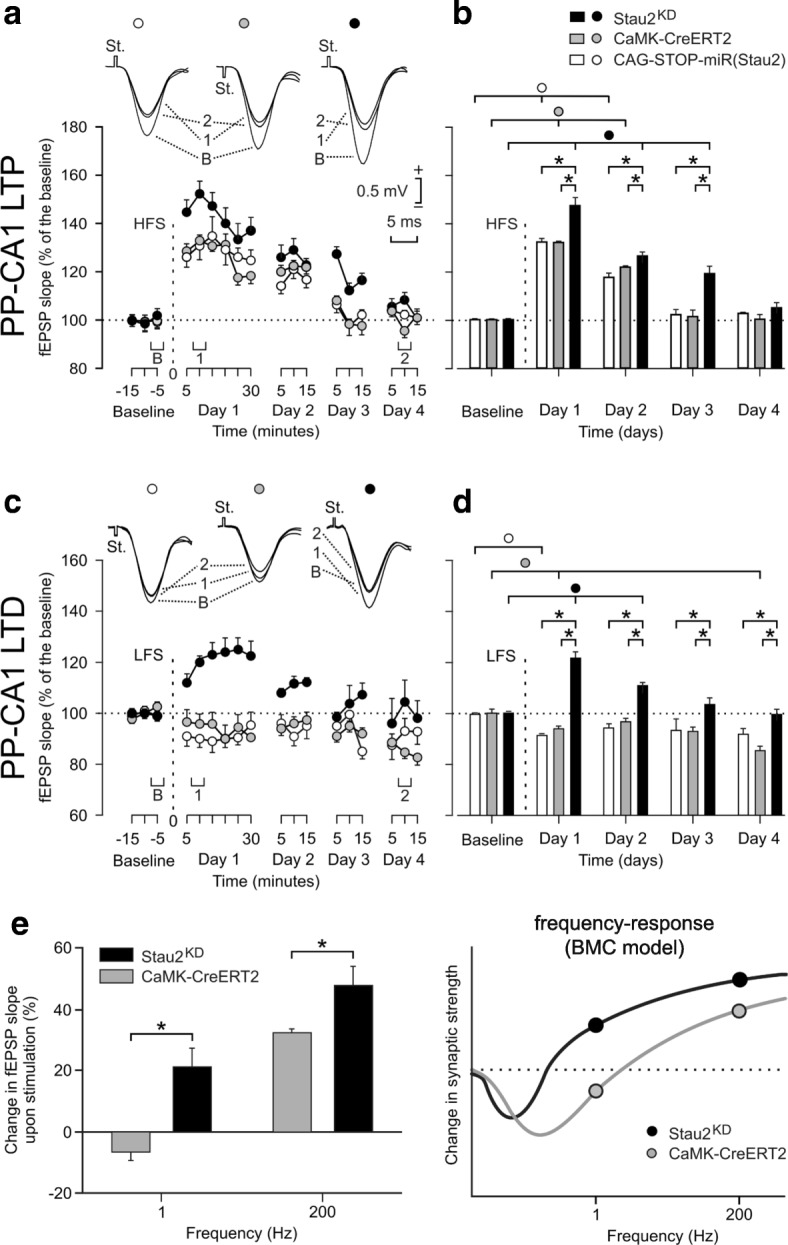
Stau2 deficiency leads to synaptic strengthening. **a**–**d** Representative fEPSPs (**a**, **c**, *top*) and averaged potentiated responses (**a** + **c**, *bottom*) following 600 pulses at 200 Hz (high-frequency stimulation [HFS], **a**) or 900 pulses at 1 Hz (low-frequency stimulation [LFS], **c**) of the PP in Tx-injected CAG-STOP-miR(Stau2) (*white*), CaMKIIα-CreERT2 (*gray*), or Stau2^KD^ rats (*black*) recorded in CA1. Displayed fEPSPs were taken at time points B, 1, and 2 indicated in the figure. Quantitative analysis of fEPSPs following HFS (**b**) or LFS (**d**). **e** Averaged potentiated responses recorded in CA1 on day 1 following stimulation with either LFS or HFS (*left*) and illustration of postulated frequency-response functions of hippocampal synaptic plasticity in CaMKIIα-CreERT2 (*gray*) and Stau2^KD^ rats (*black*) according to the BCM frequency-response function for the data points recorded in CA1 (*right*). Statistical significant differences within groups are indicated using *horizontal bars* with *circles*, and between groups with *stars* after two-way ANOVA of repeated measures and all pairwise multiple comparison procedures (Holm–Sidak method) (n = 10 animals/group). *,○ *p* < 0.05

LTP was induced by applying a high-frequency stimulation (HFS) protocol (five 200 Hz, 100 ms trains of pulses at a rate of 1/s; presented six times, at intervals of 1 min) onto PP projections in Tx-treated rats of the three genotypes while recording at CA1 and CA3 sites. HFS led to a significantly higher potentiation of evoked fEPSPs in CA1 (Fig. 2a, b) and CA3 (Additional file 1: Figure S2E, F) in Stau2^KD^ rats than in both control animals. LTP in Stau2^KD^ rats lasted longer in both CA1 (72 h; Fig. 2b) and CA3 (96 h; Additional file 1: Figure S2F), whereas it decayed significantly faster in both Tx-treated control animals. Moreover, as there were no differences detected between both control lines, we can neglect possible side effects due to unregulated recombination of the transgene in the CAG-STOP-miR(Stau2) rat line in the hippocampus.

For the induction of LTD, Tx-treated animals were exposed to a low-frequency stimulation (LFS) protocol (900 pulses at 1 Hz) on PP synapses after appropriate baseline recordings in CA1 (Fig. 2c, d) and CA3 sites (Additional file 1: Figure S2G, H). Both control animals showed a significant depression of evoked responses lasting for at least 24 h, while Stau2^KD^ rats did not show LTD but rather LTP in both CA1 and CA3, persisting up to 72 h post stimulation. Again, both control animals behaved similarly.

Together, our experiments show increased LTP at HFS and the induction of LTP instead of LTD at LFS at hippocampal synapses in Stau2^KD^ animals (Fig. 2e), suggesting a shift in the frequency-response function of long-term synaptic plasticity favoring synaptic strengthening. Similar observations have been reported for mutant PSD-95 mice [23]. This alteration is consistent with the Bienenstock, Cooper, and Munro (BCM) theory of synapse modification [24] that proposes a sliding threshold for the induction of LTP and LTD in response to different stimulating frequencies. Consequently, this proposed shift in synaptic strength as shown in Fig. 2e (right panel) represents our current working model. The general enhancement of synaptic strength upon stimulation in the Stau2^KD^ rats extends previous findings reporting that Stau2 knockdown in brain slices impairs mGluR-induced protein synthesis-dependent LTD [13]. Together, we established a functional contribution of the RBP Stau2 in distinct aspects of synaptic plasticity.

### Stau2^KD^ rats displayed deficits in spatial short-term memory and spatial working memory

**Fig. 3:**
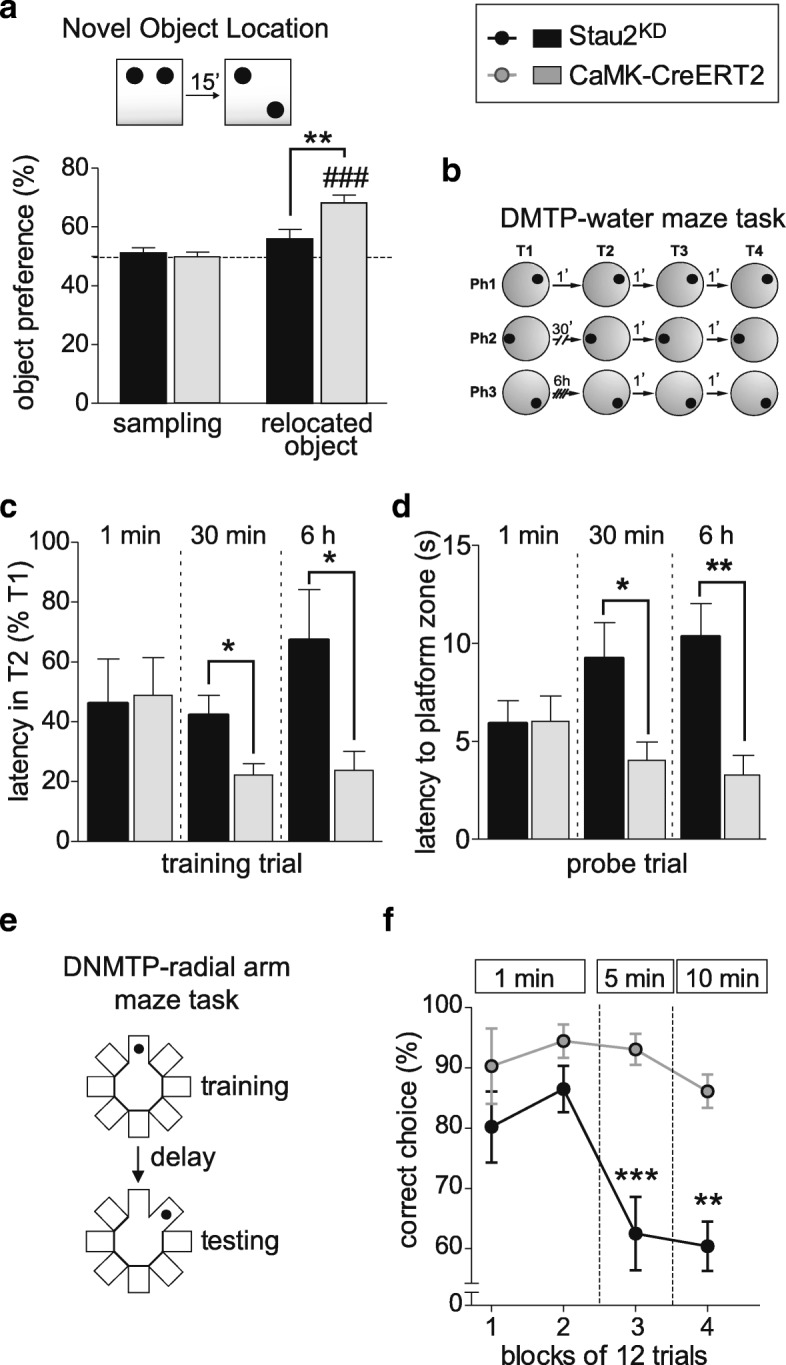
Defective spatial novelty detection and spatial working memory in Stau2^KD^ rats. **a** NOL task is schematically represented (*top*); preference for the novel object in the hippocampus-dependent NOL task for Tx-treated Stau2^KD^ (*black*; n = 12) and CaMKIIα-CreERT2 (*gray*; n = 8) rats (NOL: F_*genotype*_ (1,18) = 4.47; *p* = 0.049). Values > 50% indicate that the animals spent more time investigating the novel object during the study phase. **b**–**d** DMTP water maze task. *Schematic representation* of the DMTP task (**b**), consisting of four trials each day (T1–T4). Within a particular day, the position of the escape platform (*dot*) was fixed, but changed to a different position between days. During initial training and testing, the delay interval between all daily trials was 1 min (Ph 1). Subsequently, only the delay interval between T1 and T2 was extended to 30 min (Ph 2) or 6 h (Ph 3). Latency in T2 to find the escape platform, expressed as percentage of the initial training trial T1 (**c**) and the latency to reach the platform zone (platform plus surrounding area) during the probe trial on T2 (**d**) for CaMKIIα-CreERT2 and Stau2^KD^ animals (n = 12 animals/group). **e**, **f** *Schematic representation* of DNMTP eight-arm radial maze task (**e**). *Plots* indicating the percentage of animals that make the correct arm choice during the choice phase of the DNMTP task after short (1 min) and intermediate (5 min, 10 min) time delays for CaMKIIα-CreERT2 (n = 6) and Stau2^KD^ animals (n = 8) (F_*genotype*_ (1,36) = 21.12; *p* < 0.001). Figures show mean + or ± SEM. *Stars* represent *p* values between genotypes obtained by either t-test (**a**, **c**, **d**) or Bonferroni post hoc analysis following two-way ANOVA of repeated measures (**f**). *Hashtags* represent *p* values of one-sample t-tests to chance level 50%: * *p* < 0.05; ** *p* < 0.01; ***,### *p* < 0.001

As changes in hippocampal LTP and LTD generally correlate with behavioral alterations in various spatial learning and memory tasks [25], we decided to investigate these cognitive tasks in Stau2^KD^ rats. Since Tx-treated CaMKIIα-CreERT2 and CAG-STOP-miR(Stau2) rats displayed equivalent electrophysiological properties (Fig. 2, Additional file 1: Figure S2) and to avoid artefacts due to Tx-treatment per se [26–29], we decided to use Tx-treated CaMKIIα-CreERT2 animals as controls in behavioral experiments (referred to as CaMKIIα-CreERT2). Basal locomotor activity of Stau2^KD^ rats was indifferent to CaMKIIα-CreERT2 animals (Additional file 1: Figure S3A–D). We performed behavioral analyses to assess short-term memory formation. Both groups discriminated similarly a novel object from a familiar object in the novel object recognition (NOR) task (Additional file 1: Figure S3E). In the hippocampus-dependent novel object location (NOL) paradigm, however, Stau2^KD^ animals failed to recognize the novel position of a familiar object in contrast to CaMKIIα-CreERT2 rats (Fig. 3a), suggesting that Stau2^KD^ rats have a specific deficit for spatial novelty detection. Next, we assessed the ability for spatial reference learning and memory. In the hidden platform version of the Morris water maze, both Stau2^KD^ and CaMKIIα-CreERT2 rats learned to find the escape platform equally well (Additional file 1: Figure S3F). On a probe trial with the escape platform removed, both groups spent similar time investigating the target quadrant (Additional file 1: Figure S3G), suggesting that Stau2^KD^ rats have unaltered spatial reference memory. We tested spatial working memory by performing a delayed matching to place task (DMTP) in the water maze and a delayed non-matching to place task (DNMTP) on an eight-arm radial maze [30–32]. In the DMTP paradigm, rats had to locate a hidden escape platform, which position remained constant on four consecutive trials per day, but was changed between days (Fig. 3b). Remembering the recent platform location could be inferred from a decrease in escape latency between trials. When the time interval between all daily trials was uniformly 1 min, both Stau2^KD^ and CaMKIIα-CreERT2 rats performed equally well (Fig. 3c). However, once the time interval between the first (T1) and the second (T2) trial was increased to either 30 min or 6 h, Stau2^KD^ animals required a significantly longer time to find the correct platform position on T2 (Fig. 3c). When animals were subjected to probe trials, in which the escape platform was removed only on T2, Stau2^KD^ rats also displayed a distinct deficit in memorizing the platform zone when the time interval between T1 and T2 increased to 30 min or 6 h (Fig. 3d), indicating that Stau2^KD^ had impaired memory of the precise spatial location of the recent escape platform. Whether these observed deficits were due to either a lack of improvement throughout the entire task or stand for a net deficit in spatial working memory need further investigation. Similar results were obtained when animals were tested in the DNMTP task, where animals were trained to distinguish an unvisited arm of the radial maze from a previously visited one in order to be rewarded. Remembering the visited arm was made gradually more difficult by introducing a delay time between the initial visit (training) and the choice phase (testing), during which the animal had no possibility to explore the maze (Fig. 3e). While both groups displayed similar accuracies in collecting rewards at 1-min delay, Stau2^KD^ rats significantly dropped in their performance at intermediate delay times (5 or 10 min) (Fig. 3f), without showing an increased impulsivity (Additional file 1: Figure S3H). These findings are consistent with a hippocampus-dependent deficit [30]. Moreover, Stau2 deficiency impaired behaviors that depend on LTD formation, i.e. spatial short-term memory and spatial working memory [33, 34], hence correlating defective behavior with the LTD deficits observed in the Stau2^KD^ rats. Classic studies have correlated hippocampal integrity [31] or changes in hippocampal LTP [32] with behavioral differences in spatial reference memory. Recent studies, however, investigating conditional mouse models with a very specific hippocampal NMDA receptor deficiency demonstrated a more complex role for the hippocampus, as spatial reference memory remained intact, but the spatial working memory was impaired in these knockout mice [35]. Thus, we speculate that Stau2 plays an important role in setting the threshold in hippocampal-specific synaptic plasticity processes, as enhanced and longer-lasting hippocampal LTP in Stau2^KD^ rats led to rather specific deficits in spatial working memory without affecting spatial reference memory.

### Stau2^KD^ rats displayed deficits in temporal and spatial association memory

We then tested Stau2^KD^ rats in tasks assessing conditioned fear learning and memory. Stau2^KD^ and CaMKIIα-CreERT2 rats were equivalent in fear learning, hippocampus-dependent contextual fear memory and hippocampus-independent fear memory for the cue within a delay fear conditioning task (Additional file 1: Figure S4A–C), and in the extinction of the learned fear association (Additional file 1: Figure S4D).

**Fig. 4:**
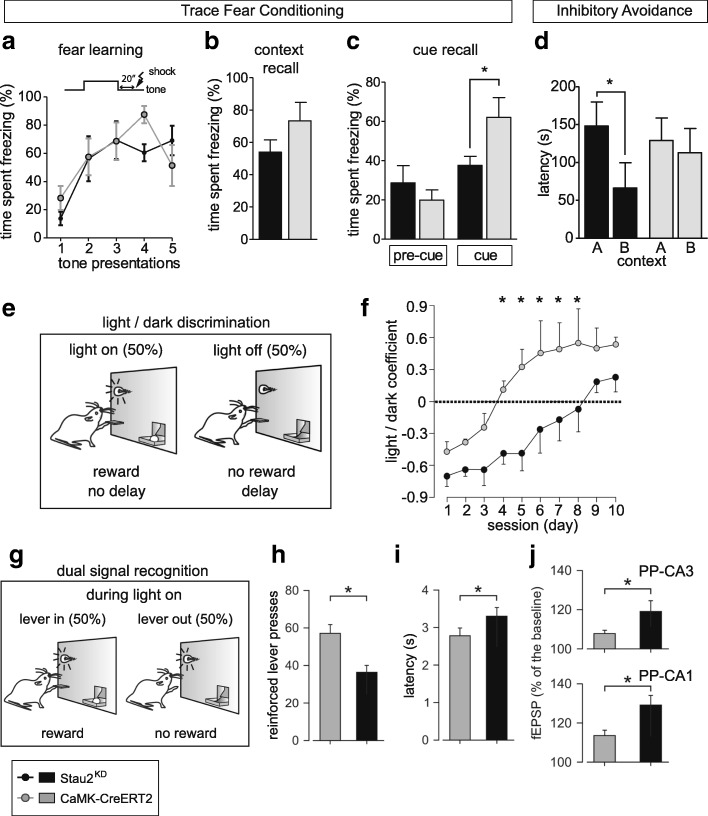
Selective deficits in associative fear memory and operant conditioning in Stau2^KD^ rats. **a**–**c** Trace fear conditioning. *Schematic representation* of the training test is indicated (**a**; *top*). Acquisition of conditioned fear shown as time spent freezing plotted over tone-shock pairings (**a**; *bottom*), recall of contextual fear displayed as freezing response toward the context in which fear conditioning took place (**b**) and freezing response toward the presented tone cue (**c**) in Tx-treated CaMKIIα-CreERT2 and Stau2^KD^ animals (n = 7 animals/group). **d** Inhibitory avoidance task. Latency to step down the platform with all four paws was recorded for Stau2^KD^ (*black*; n = 10) and CaMKIIα-CreERT2 (*gray*; n = 11) rats both in the original conditioning chamber A and the testing chamber B. **e** *Illustration* of the light/dark discrimination task, where lever presses during light periods were rewarded, while lever presses in the dark were not rewarded and led to a 10-s delay for the reappearance of the light. **f** Stau2^KD^ animals displayed significant deficits to newly introduced association of the light cue with lever presses being rewarded, as expressed by the change in the light/dark coefficient over training days. Positive values indicate a preference of rewarded lever presses in the light period over non-rewarded lever presses in the dark period (n = 10 animals/group). **g** *Schematic representation* of the dual signal recognition task, where the lever (and reward) was removed in 50% of the illuminated periods. **h**, **i** Adaptation of the CaMKIIα-CreERT2 controls and Stau2^KD^ rats to the dual signal recognition task (**h**) and latency to approach the lever during light periods (**i**). **j** Slopes of fEPSPs evoked at the PP-CA3 (*top*) and PP-CA1 (*bottom*) synapses, recorded when the CaMKIIα-CreERT2 controls and Stau2^KD^ rats were crossing the photoelectric beam 1 (n = 10 animals/group). *Stars* represent *p* values between genotypes obtained by t-tests (**c**, **d**) or two-way ANOVA of repeated measures and all pairwise multiple comparison procedures (Holm–Sidak method; **f**, **h**–**j**). * *p* < 0.05

To test for hippocampal deficiencies in temporal associative fear learning and memory, Stau2^KD^ and CaMKIIα-CreERT2 rats were subjected to a trace fear conditioning task (Fig. 4a–c), in which the shock was administered 20 s after termination of each tone presentation. Both genotypes were indistinguishable in the development of the fear response towards the cue (Fig. 4a) and in the fear response to the context (Fig. 4b). However, Stau2^KD^ rats displayed a significantly attenuated fear response towards the cue compared to CaMKIIα-CreERT2 animals (Fig. 4c), indicating a deficiency to associate the tone with the non-simultaneous shock.

In order to study spatial association learning and memory, we conducted an inhibitory avoidance task. Rats of both genotypes received an electric shock once they stepped down from a platform onto the shock grid in a conditioning chamber “A” (Additional file 1: Figure S4E). The following day, trained animals were tested first for their latency to step onto the shock grid in the original conditioning chamber “A” and subsequently in a slightly modified testing chamber “B” (Additional file 1: Figure S4F). CaMKIIα-CreERT2 rats showed a similar step-down latency in both tested contexts (Fig. 4d), indicating that the animals recognized commonalities of both contexts (i.e. the shock grid) and associated them with the aversive experience [36]. Stau2^KD^ rats, however, displayed a significantly reduced step-down latency in the testing chamber “B” compared to the conditioning “A” (Fig. 4d), suggesting that they have impaired fear memory for the second context and a deficit in spatial association memory. This suggests that Stau2 is required for both spatial and temporal association fear memory, which, similar to working memory, is dependent on the connectivity of medial prefrontal cortex and hippocampus [37].

### Stau2^KD^ rats displayed deficits in associative learning in operant conditioning

Hippocampus-dependent synaptic plasticity differentially contributes to associative learning in operant conditioning paradigms as well [38]. Stau2^KD^ and CaMKIIα-CreERT2 rats learned to press a lever for food reward with comparable efficiency (Additional file 1: Figure S4G–I). Once stable performance had been reached, rats were then trained on a light/dark paradigm, in which only lever presses during lighted periods were rewarded while lever presses in the dark introduced a delay in the presentation of the lever (Fig. 4e). Stau2^KD^ rats learned the new protocol significantly slower than control animals (Fig. 4f) and this impaired performance was not caused by an increased impulsive behavior, since the total number of lever presses of Stau2^KD^ animals was lower than in CaMKIIα-CreERT2 controls (Additional file 1: Figure S4J).

Next, we investigated how Stau2^KD^ rats performed in a dual signal recognition task using a Skinner box modified for in vivo electrophysiological recordings during behavioral tests (Figure S4K). Animals learned that the light signal was not warranting the reward, since the lever was retracted from the box every second light period (Fig. 4g). Stau2^KD^ rats displayed a significant deficit in adapting to the new protocol in comparison to control animals (Fig. 4h and data not shown), without showing an impulsive choice (Fig. 4i). When approaching the lever, Stau2^KD^ animals displayed a significantly higher increase of evoked fEPSP slopes compared to control rats (Fig. 4j and data not shown).

## Conclusions

Stau2^KD^ animals showed a series of learning deficits including impairments in spatial short-term and spatial working memory, spatial and temporal association fear memory, as well as an inability to acquire novel associations to be rewarded in complex operant conditioning tasks. Importantly, in vivo electrophysiological recordings allowed us to detect Stau2-dependent changes of synaptic plasticity in behaving animals (Fig. 4j). Taken together, we demonstrate that Stau2 downregulation impairs the flexibility of the animals in adapting to a new condition in order to receive a reward. Interestingly, parallel recording in freely behaving animals detected enhanced LTP together with impaired LTD. We hypothesize that the changes in behavior might be correlated with the shift in the frequency-response function of synaptic plasticity. Together, our molecular, morphological, electrophysiological, and behavioral findings have several important implications for delineating the role of the dsRBP Stau2 in synaptic function and region-specific behavior and circuitry. Previous reports with conditional mouse mutants showed that LTD impairment led to comparable spatial learning and memory phenotypes as observed in Stau2^KD^ rats, although the LTD deficits were not correlated with a shift in the BCM frequency-response function [33, 34, 39]. Furthermore, in vivo studies demonstrated that LTD might underlie the encoding of object-place configuration in rats [40]. We speculate that Stau2-mediated LTD deficits are responsible for the observed cognitive changes. Future experiments will have to unravel the detailed role of Stau2 in different phases of learning and memory. Finally, it will be important to delineate the precise synaptic connections that are involved in the modulation of hippocampus-dependent behavioral traits. For instance, the entorhinal cortex (EC) projects to either the CA1 area or the DG in the hippocampus [41]. Selective blocking of synaptic transmission within the pathway from EC to CA1 by either complex transgenic mouse models [42] or local injection of dopamine-receptor agonists into CA1 [43], causes deficits in spatial short-term memory and spatial working memory. Interestingly, several components of the dopaminergic signal transduction pathway are physiological mRNA targets of Stau2 [15, 44], raising the possibility that Stau2 affects these responses thereby modifying dopaminergic signal transduction.

Together, we show that the RBP Stau2 plays an important role in the regulation of specific aspects of hippocampal synaptic plasticity and learning and memory. Based on these exciting new functions of Stau2 in associative learning, we envision that Stau2 might also contribute to synaptic tagging and capture [45, 46] as outlined in our running sushi belt model [47]. We think that Stau2^KD^ rats represent an important tool to study specific aspects of synaptic plasticity, particularly involving physiological and pathological forms of short-term, working memory [3], and even episodic-like memory [48].

## Methods

### Generation and maintenance of transgenic rats

The expression vector pCAG-loxP.STOP.loxP-miR(Stau2)-EGFP (CAG-STOP-miR(Stau2); Fig. 1a) is based on the plasmid CAG-loxP.EGFP [20] by changing the ORF of the *loxP*-flanked *lacZ* gene to *mCherry* (“ORF-STOP cassette”) and by inserting an artificial intron (derived from pIntron [18]) including the miR(Stau2) at the 5′-end of EGFP coding sequence. The miRNA targeting *Stau2*, miR(Stau2), generated according to the miRNA3 design [18], contains the target side of a potent siRNA inhibiting Stau2 protein production [12]. Experiments were carried out with male rats obtained by crossing the line CaMKIIα-CreERT2 #327 [20] with the line CAG-STOP-miR(Stau2) #17. At the age of ten weeks, double transgenic rats were intraperitoneally injected with tamoxifen (40 mg/kg, seven injections/week, at least ten days before starting the experiments) to obtain Stau2 knockdown (Stau2^KD^) rats. DNA sequences are available upon request.

For constitutive and ubiquitous miR(Stau2) expression the CAG-STOP-miR(Stau2) rats were bred with the Cre-deleter rat line. In brief, an EF1α-Cre construct was targeted to the rat ROSA26 locus using zinc finger nuclease-mediated gene targeting, by combined mRNA and plasmid microinjections in fertilized oocytes. The resulting offspring expresses the Cre-recombinase in the rat germline which leads to a complete recombination of floxed transgenes in Cre-positive animals (Stau2-silenced). A detailed description of this rat line is available upon request.

### Molecular biology

#### Quantification of RNA concentrations by real time RT-PCR

To determine the mRNA concentrations of genes displayed in Additional file 1: Figure S1D–F, hippocampal areas were microdissected from thick frozen brain slices. Total RNA was isolated using TRIzol Reagent (Thermo Scientific) according to the manufacturer’s protocol. Complementary DNA (cDNA) was synthesized from 0.5–1 μg RNA. Quantification of respective mRNAs was conducted as described [18] with slight modifications. Detection of processed miRNA was performed using a Custom Taqman Small RNA Assay (Applied Biosystems, #ID: CS70K4Q) according to the manufacturer’s instructions. All real-time PCR reactions were run on a 7900 HT fast real-time PCR system (Applied Biosystems).

To determine mRNA concentrations of the genes displayed in Fig. 1f, forebrains were collected and snap-frozen. Total RNA was isolated using TRIzol Reagent, followed by DNase treatment in column (Qiagen). cDNA was synthesized from 0.5–1 μg DNase-treated RNA using random primers and SuperscriptIII™ reverse transcriptase (Invitrogen) according to the manufacturer’s instructions. To avoid contamination with genomic DNA, DNase-treated RNA was split in two reactions: one containing reverse transcriptase and the other without the enzyme. Both were run in parallel in qRT-PCR using SYBR green mastermix, in a Light Cycler 96 (Roche). Primers were previously reported and optimized to achieve 95–105% efficiency [15]; qRT-PCR data were analyzed using the comparative ΔΔCT method [49]. Oligonucleotide sequences are included in Additional file 2.

#### Quantification of transgene copy number

Copy number quantification per cell was done by genomic qRT-PCR as previously described [21], using tail DNA from CAG-STOP-miR(Stau2) and CAG-STOP-miR(Stau2) × Cre-deleter animals. Experimental details and oligonucleotide sequences are included in the Additional file 2.

#### Western blots

Dissected tissues were homogenized and equivalent amounts of protein were separated via 10% SDS-PAGE and subjected to immunoblotting. Membranes were blocked using 2% BSA in TBS/0.1% Tween-20. Membranes were incubated with the respective primary antibodies (see the Additional file 2) overnight at 4 °C, and the IRDye800 labeled secondary antibodies for 1 h. Membranes were scanned with the infrared-based Odyssey Imaging System (Li-Cor) and quantified using the Image Studio software.

### Microscopy

#### Immunohistochemistry and immunofluorescence

Dissected brains from perfused animals were postfixed with 4% paraformaldehyde (PFA) in PBS at 4 °C for 24–48 h and brain sections (50 μm) yielded by using a vibratome (Leica, Germany). Neurons in culture were fixed with warm 4% PFA for 15 min. Immunohistochemistry using DAB staining was performed as described [50] with slight modifications. Double immunofluorescence staining was performed as described [20]. Stained sections were examined either with a Zeiss Stemi 2000C or a Leica SP5 confocal laser-scanning microscope. For detailed experimental procedures and antibodies, see Additional file 2.

#### In situ hybridization

In situ hybridizations (ISH) were made as previously described [51], using DIG-labeled antisense probes directed against the intron of the long isoform of rat *Calm3* [22] or sense probes as control on PFA fixed free-floating vibratome sections. Stained sections were examined with a Zeiss Axio Imager M2 microscope using 10× magnification and the tiling mode of ZEN software. Tiles were stitched with the ImageJ Plugin Stitch Multiple Series or Tile Scan File [52].

#### Analysis of dendritic spines

Golgi impregnations were performed using FD GolgiStain Kit (FD NeuroTechnologies, USA) and analyzed as described [53]. Experimental details are described in Additional file 2.

### Electrophysiology and behavioral tests

#### Electrophysiological in vivo recordings

Surgical implantation of stimulating and recording electrodes as well as stimulating and recording procedures for basal synaptic transmission, paired pulse facilitation, LTP (600 pulses at 200 Hz), and LTD (900 pulses at 1 Hz) were conducted as described [54, 55] with slight modifications. Detailed experimental procedures are described in Additional file 2.

#### Behavioral analysis

Males of both double transgenic and control animals aged 2–3 months were injected with Tx [20]. All the behavioral tests were performed as previously described, with slightly modifications: the open field task, the novel object recognition test, and the novel object location test [19]; delay and trace fear conditioning [56]; Morris water maze task to test for spatial reference memory [57]; DMTP in the water maze [58]; DNMTP on the eight-arm radial maze [30]; inhibitory avoidance task [59]; and operant conditioning paradigms [38]. Detailed experimental procedures are described in Additional file 2.

### Statistical analysis

Statistical analyses were performed using either the t-test, univariate or multivariate analysis of variance (ANOVA) with repeated or independent measures, followed by either a Bonferroni post hoc test or contrast analysis (Holm–Sidak method) to determine significant differences. Respective F- and *p* values were calculated using either GraphPad Prism 5.0 or SPSS Version 18. All data are presented as either mean + SEM or mean ± SEM, unless stated otherwise. *p* < 0.05 was considered statistically significant.

## Additional files

Additional file 1: Figure S1. Characterization of conditional, forebrain-specific Staufen2 knockdown rat. **Figure S2.** Stau2 deficiency leads to a shift in the frequency-response function of hippocampal synaptic plasticity favoring synaptic strengthening. **Figure S3.** Consequences of Stau2 knockdown on hippocampal-dependent spatial learning and memory. **Figure S4.** Consequences of Stau2 knockdown on fear learning, memory, and operant conditioning. (PDF 3036 kb)

Additional file 2: Supplemental experimental procedures and references. (DOCX 69 kb)
