# Supplementary material for: Forebrain-specific, conditional silencing of Staufen2 alters synaptic plasticity, learning, and memory in rats
Source: Genome Biol. 2017 Nov 17;18:222. doi: 10.1186/s13059-017-1350-8 (PMC5693596; doi:10.1186/s13059-017-1350-8)
Supplement: Supplementary file 1 — Characterization of conditional, forebrain-specific Staufen2 knockdown rat. Figure S2. Stau2 deficiency leads to a shift in the frequency-response function of hippocampal synaptic plasticity favoring synaptic strengthening. Figure S3. Consequences of Stau2 knockdown on hippocampal-dependent spatial learning and memory. Figure S4. Consequences of Stau2 knockdown on fear learning, memory, and operant conditioning. (PDF 3036 kb) [file 13059_2017_1350_MOESM1_ESM.pdf]

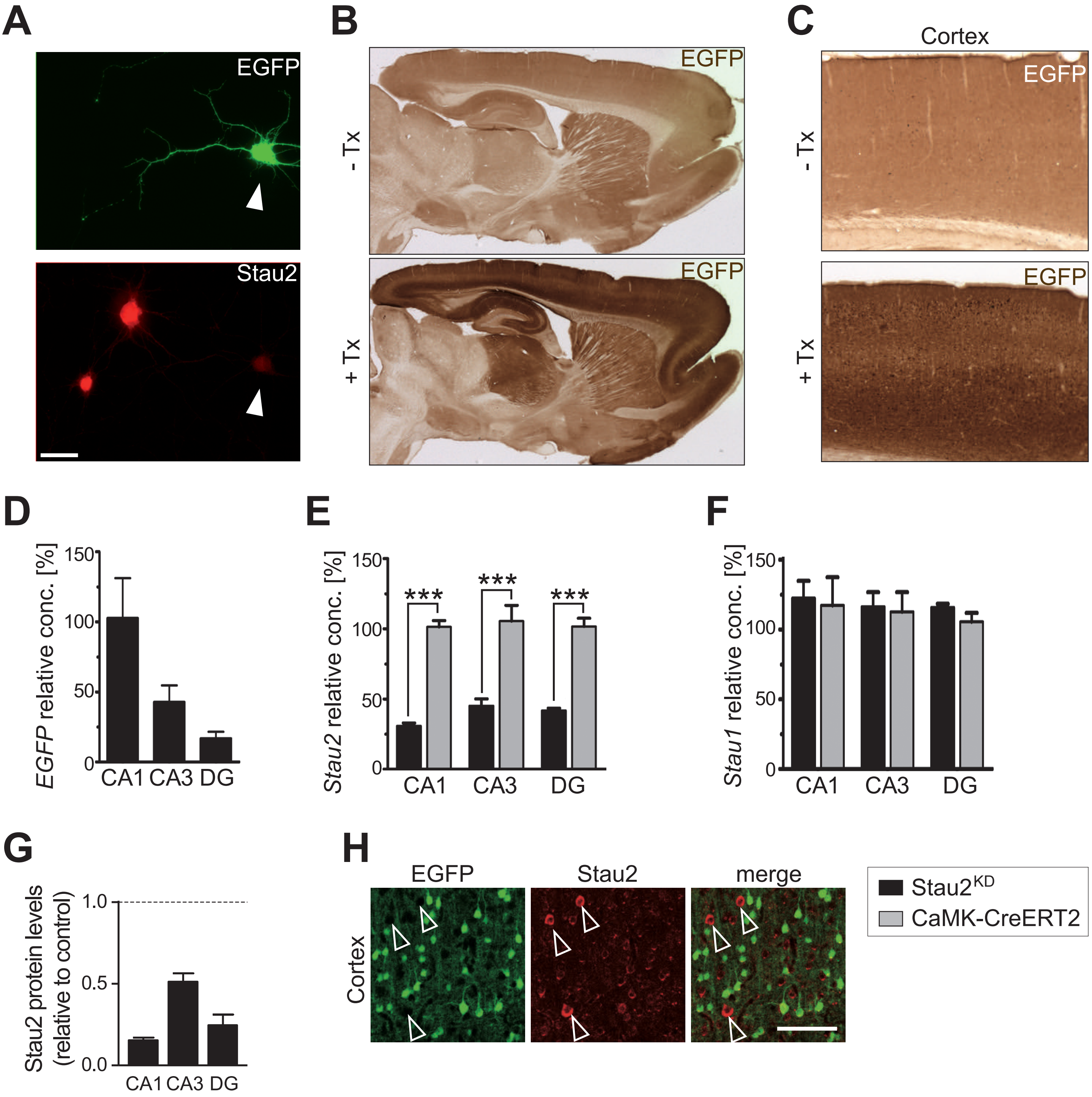

Berger et al. **- Figure S1 -**

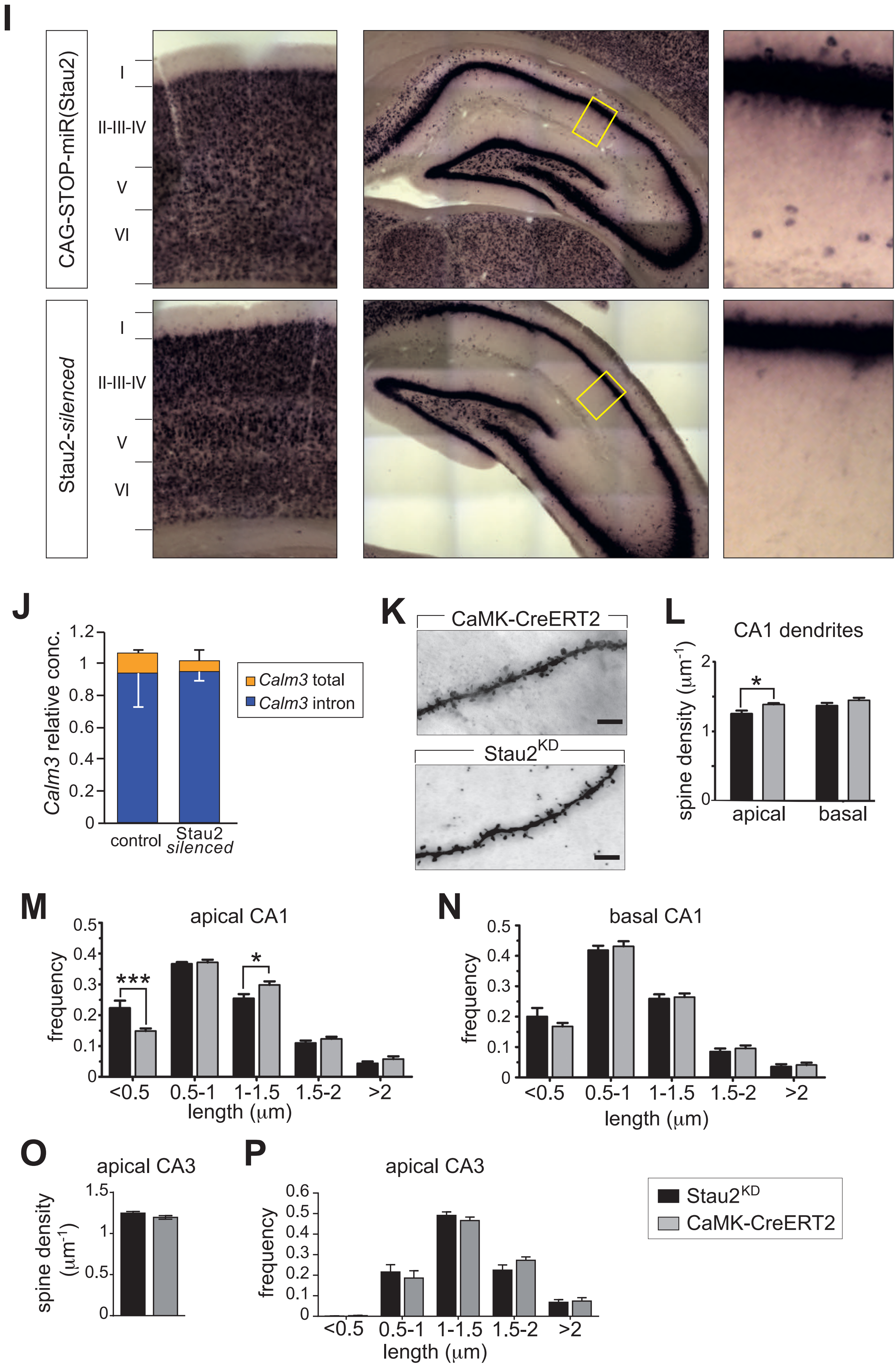

Berger et al. - **Figure S1 (continued) -**

Figure S1: Characterization of conditional, forebrain-specific Staufen2 knockdown rat (related to **Figure 1**).

(**A**) Immunostaining anti-Stau2 (Cy5; red) of primary hippocampal neurons, transiently transfected with a vector co-expressing EGFP (green) and miR(Stau2). Arrowhead indicates a transfected EGFP positive neurons that show almost no signal for Stau2. (**B-C**) Immunohistochemical analysis for EGFP in forebrain (**B**) and cerebral cortex (**C**) of double transgenic CaMKIIα-CreERT2 x CAG-STOP-miR(Stau2) rats following vehicle (-Tx) or tamoxifen (+Tx; Stau2^{KD}) injection. (**D-F**) Quantification by qRT-PCR of *EGFP* (n=4 animals/group) (**D**), *Stau2* (n=5 animals/group) (**E**) and *Stau1* (n=5 animals/group) (**F**) mRNA expression in hippocampal areas of Stau2^{KD} animals (black bars), and compared to CaMKIIα-CreERT2 animals (gray bars) (**E**: $F_{genotype}$ (1,24) = 172.18; p < 0.001). (**G**) Quantification of Stau2 protein levels relative to control (as in **Figure 1D**) in Stau2^{KD} **(**n=3 animals/group). (**H**) Dual immunofluorescence visualization of EGFP (Cy2; green) and Stau2 (Cy5; red) within the cerebral cortex; arrowheads indicate Stau2-positive immunostaining in EGFP-negative neurons. (**I-J**) Analysis of the intron-containing *Calm3* mRNA isoform in control and Stau2 silenced animals: representative images of *in situ* hybridization with *Calm3* intron antisense probe in free-floating adult coronal vibratome sections, showing cortex (left panels) and hippocampus (middle panels, yellow boxes are magnified on the right panels) (**I**) and qRT-PCR of total *Calm3* mRNA and *Calm3* containing intron in cortical lysates. Bars show mean and SD values from n=3-4 animals/group (**J**). (**K-P**) Effect of Stau2 deficiency on dendritic morphology *in vivo*. (**K**) Representative Golgi-Cox staining of a CA1 dendrite from Tx-injected CaMKIIα-CreERT2 (left) and Stau2^{KD} (right) rats. Quantification of dendritic spine density (**L,O**) and frequency distribution of dendritic spine length in apical (**M,P**) and basal (**N**) dendrites of CA1 (**L-N**) or CA3 (**O,P**) pyramidal neurons in Stau2^{KD} rats compared to CaMKIIα-CreERT2 rats (CA1: $F_{genotype*genotype}$ (4,32) = 6.32; p = 0.0007) (n=4-5 animals/group). Figures show mean + SEM. Scale bars: 20μm (**A**), 100μm (**H**), 10μm (**K**). Stars represent p-values between genotypes obtained by t-tests (**E,L**) or Bonferroni post-hoc analysis following two-way ANOVA of repeated measures (**M**); * p<0.05; *** p<0.001.

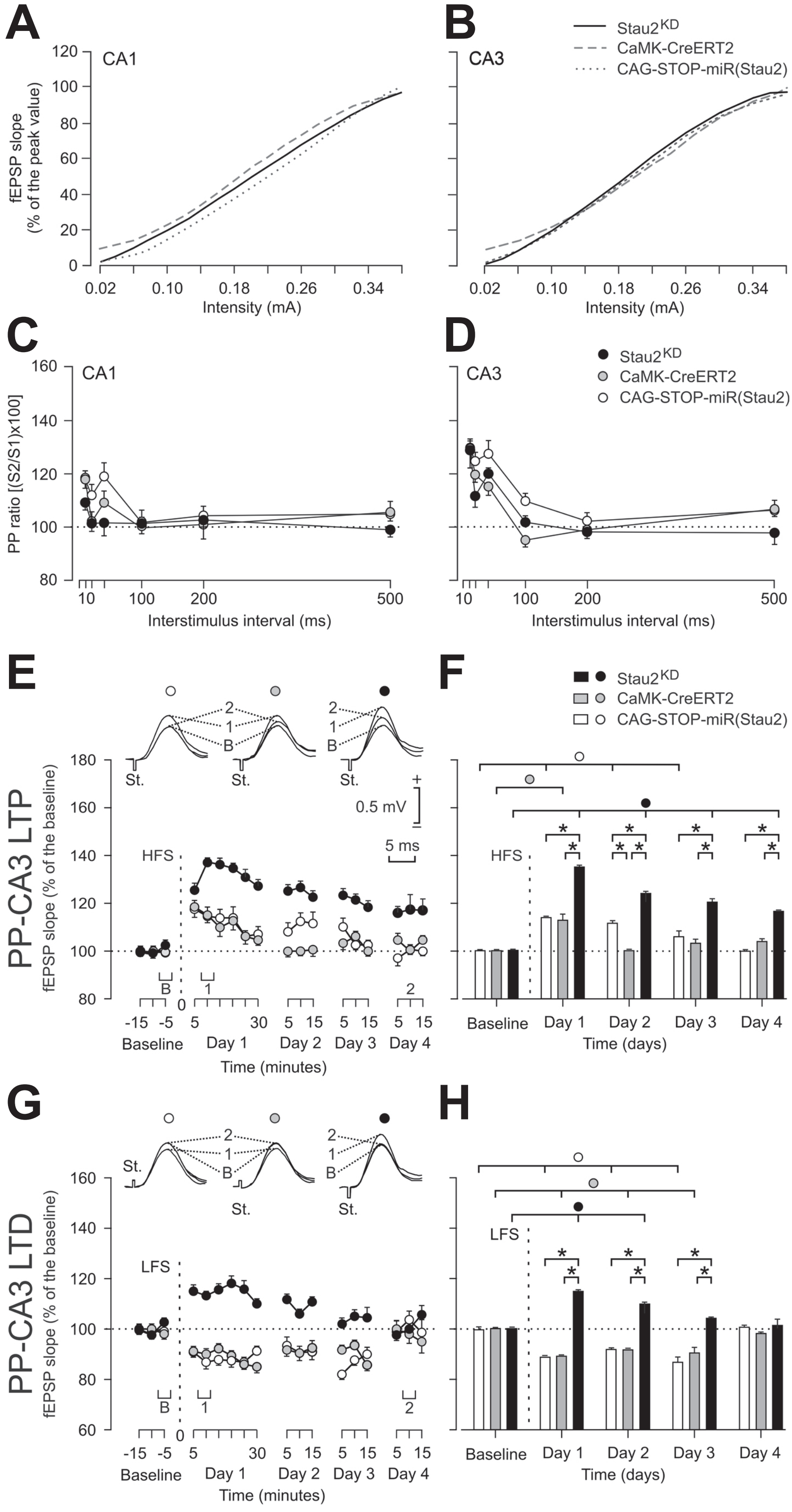

Berger et al. **- Figure S2 -**

Figure S2: Stau2 deficiency leads to a shift in the frequency-response function of hippocampal synaptic plasticity favoring synaptic strengthening (related to **Figure 2**).

(**A-B**) Input-output relationship is shown as the ratio between the stimuli intensity and the evoked fEPSP slope in the CA1 (**A**) and CA3 (**B**) regions of Tx-injected CAG-STOP-miR(Stau2) (dotted line), CaMKIIα-CreERT2 (dashed line) or Stau2^{KD} (solid line) rats. (**C-D**) Paired pulse (PP) facilitation evoked by the second pulse at different stimulus intervals in the CA1 (**C**) and CA3 (**D**) regions of Tx-injected CAG-STOP-miR(Stau2) (white), CaMKIIα-CreERT2 (gray) or Stau2^{KD} rats (black). (**E-H**) Representative fEPSPs (**E+G**, top) and averaged potentiated responses (**E+G**, bottom) following high frequency stimulation (HFS, **E**) or low frequency stimulation (LFS, **G**) of the perforant path (PP) in Tx-injected CAG-STOP-miR(Stau2) (white), CaMKIIα-CreERT2 (gray) or Stau2^{KD} rats (black) recorded in CA3. Displayed fEPSPs were taken at time points B, 1 and 2 indicated in the figure. Quantitative analysis of fEPSPs following HFS (**F**) or LFS (**H**) in Tx-injected CaMKIIα-CreERT2, CAG-STOP-miR(Stau2) and Stau2^{KD} rats (**H**). Figures show mean + SEM. Statistical significant differences *within groups* are indicated using horizontal bars with circles, *between groups* with stars after two-way ANOVA of repeated measures and all Pairwise Multiple Comparison procedures (Holm-Sidak method), (n=10 animals/group). *,○ $p<0.05$.

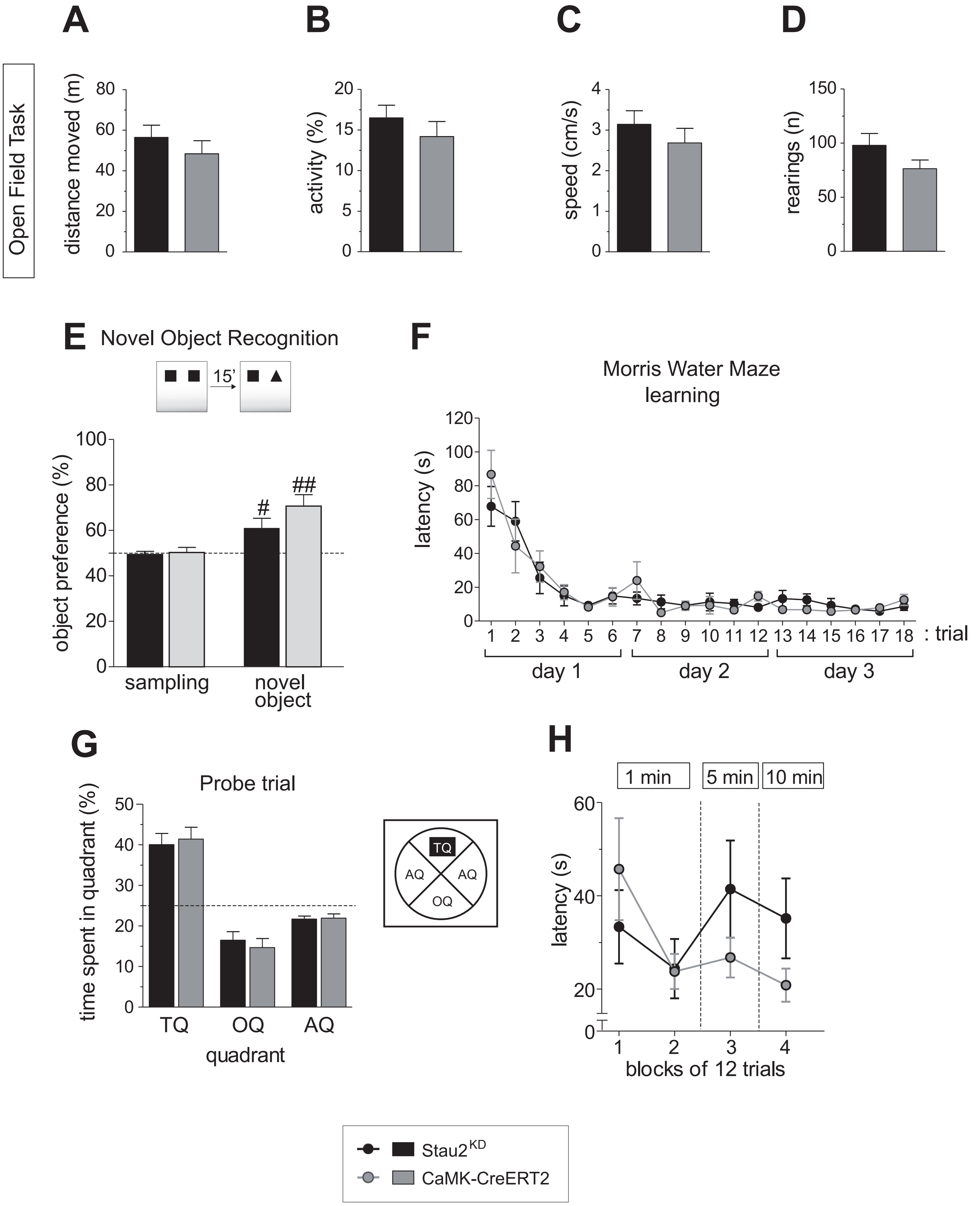

Berger et al. **- Figure S3 -**

Figure S3: Consequences of Stau2 knockdown on hippocampal-dependent spatial learning and memory (related to **Figure 3**).

(**A-D**) Open field test: total distance moved (**A**), percentage of activity (**B**), average speed (**C**) and number of rearings (**D**) during the entire test (30 min) for Tx-injected $Stau2^{KD}$ (black; n=12) and CaMKIIα-CreERT2 (gray; n=9) rats. (**E**) Novel object recognition (NOR) task is schematically represented (top), and the preference for the novel object (>50%) is indicated for Tx-injected $Stau2^{KD}$ (black; n=12) and CaMKIIα-CreERT2 (gray; n=8) rats. (**F-G**) Hidden platform Morris water maze task: averaged escape latencies over trials during training (**F**) and percentage of time spent in the different quadrants of the water maze during the probe trial on day 4 (**G**) for Tx-injected $Stau2^{KD}$ (black; n=13) and CaMKIIα-CreERT2 (gray; n=7) rats. TQ: target quadrant where the platform was located; OQ: the opposite quadrant; AQ: the average of the two adjacent quadrants. (**H**) Plot indicating the latency to enter the arm with the anticipated reward ($F_{genotype*delay\ time}(3,36) = 3.64$; $p = 0.022$) during the choice phase of the delayed non-matching to place (DNMP) task after short (1 min) and intermediate (5 min, 10 min) time delays for Tx-injected CaMKIIα-CreERT2 (n=6) and $Stau2^{KD}$ animals (n=8). Figures show mean + or ± SEM. Hashtags represent p-values of one-sample t-tests to chance level 50%: # p<0.05; ## p<0.01.

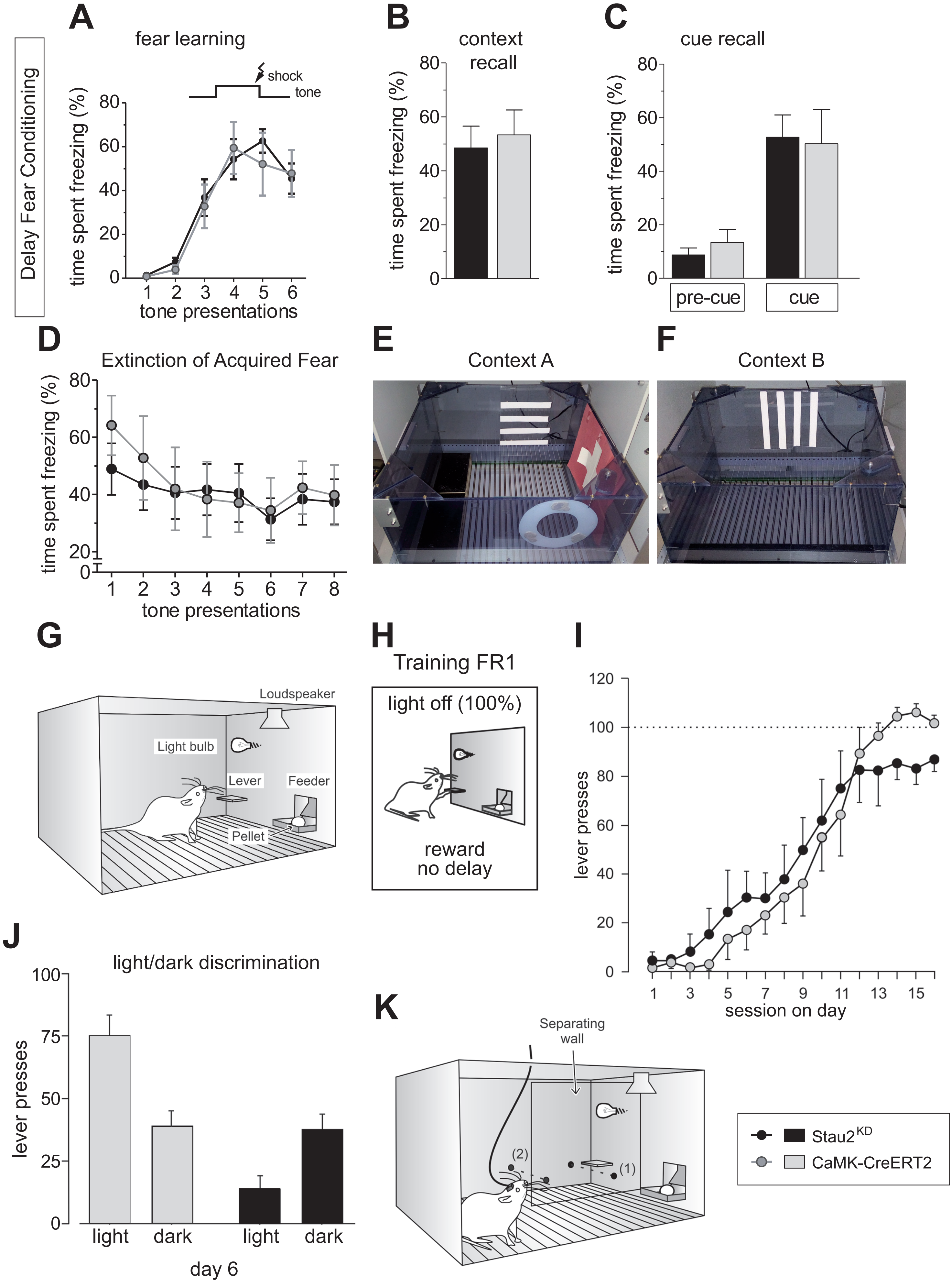

Berger et al. - **Figure S4 -**

Figure S4: Consequences of Stau2 knockdown on fear learning, memory and operant conditioning (related to **Figure 4**).

(**A-C**) Delay Fear Conditioning. Schematic representation of the training test is indicated (**A**; top). Acquisition of conditioned fear shown as time spent freezing plotted over tone-shock pairings (**A**; bottom), recall of contextual fear displayed as freezing response toward the context in which fear conditioning took place (**B**) and test for recall of cued fear, in which the freezing response was recorded in a novel context before (pre-cue) and during tone presentation (cue) (**C**) in Tx-injected CaMKIIα-CreERT2 (n=8) and $Stau2^{KD}$ animals (n=13). (**D**) Extinction of conditioned fear: time spent freezing plotted over tone presentations in the conditioning context for both Tx-injected $Stau2^{KD}$ (black; n=12) and CaMKIIα-CreERT2 (gray; n=8) rats. (**E,F**) Inhibitory avoidance task: on the training day, animals were placed on a platform located in conditioning chamber A (**E**). Once they stepped down with all 4 paws, rats received a shock and were subsequently removed from context A. 24 h later, animals were tested for their fear memory first in context A (**E**) and 3 h later in testing context B (**F**). (**G**) Schematic representation of the box used for the operant conditioning paradigm. (**H**) Illustration of the operating conditioning paradigm in the dark, applied to train the animals with a fixed ratio 1 (FR1) protocol. (**I**) Lever presses during operant conditioning session following the FR1 protocol for Tx-injected CaMKIIα-CreERT2 controls and $Stau2^{KD}$ rats. (**J**) Mean number of lever presses during light and dark periods of rats of both genotypes collected on training day 6 (as shown in **Figure 4E,F**) (n=10 animals/group). (**K**) Schematic representation of the dual signal recognition task, where the lever (and reward) was removed in 50% of the illuminated periods.
