# Supplementary material for: Forebrain-specific, conditional silencing of Staufen2 alters synaptic plasticity, learning, and memory in rats
Source: Genome Biol. 2017 Nov 17;18:222. doi: 10.1186/s13059-017-1350-8 (PMC5693596; doi:10.1186/s13059-017-1350-8)
Supplement: Supplementary file 2 — Supplemental experimental procedures and references. (DOCX 69 kb) [file 13059_2017_1350_MOESM2_ESM.docx]

**Supplemental Experimental Procedures**

***Generation of transgenic rats.*** The DNA fragment encoding miR(Stau2), inserted into the artificial intron of pIntron, has the following sequence:

5’-TGCTGTTGACAGTGAGCGAAGATATGAACCAACCTTCAAGCTGTGAAGCCACA

GATGGGCTTGAAGGTTGGTTCATATCTCTGCCTACTGCCTCGGACTTCAAGGG-3’.

For the generation of transgenic rats, the transgene encoding fragment was released by *Pme*I and *Not*I from the CAG-STOP-miR(Stau2) vector and microinjected into fertilized Sprague Dawley rat eggs at 2 ng/µl. Founder rats and their offspring were genotyped by polymerase chain reaction (PCR) on tail DNA using primers specific for EGFP. Tail DNA was yielded using the Qiagen DNeasy Blood & Tissue kit (Qiagen, Hilden, Germany). CAG-STOP-miR(Stau2) founders were crossed with the transgenic rat line CaMKIIα-CreERT2 #327 (CaMKIIα-CreERT2) [2] to obtain double transgenic CaMKIIα-CreERT2 x CAG-STOP-miR(Stau2) animals. At the age of 10 weeks, double transgenic rats were intraperitoneally injected with tamoxifen (40 mg/kg, 7 injections/week, at least 10 days before starting the experiments) to obtain Stau2 knockdown (Stau2^KD^) rats. DNA sequences and further details of the exact cloning strategy are available upon request.

Primer sequences for the genotyping PCR of EGFP to detect the CAG-STOP-miR(Stau2) construct are the following (gene sequence 5'- to 3'-):

*Gfp-genotype_fwd* ttcaaggacgacggcaactacaag

*Gfp-genotype_rev* cggcggcggtcacgaactcc

Displayed molecular, electrophysiological and behavioral experiments were carried out with male Stau2^KD^ rats obtained by crossing rats of line CaMKIIα-CreERT2 #327 with CAG-STOP-miR(Stau2) animals (line #17). Double transgenic rats and respective single transgenic controls were provided by the animal facility of the Central Institute of Mental Health (Mannheim, Germany). Animals investigated either in Mannheim or at Pablo de Olavide University in Seville (Spain) were 4 ± 1 month old upon the beginning of respective experiments. In animal facilities at both locations, rats were kept in collective cages (3-5 animals / cage) on a 12-h light/dark cycle with constant temperature (21 ± 1ºC) and humidity (50 ± 7 %) and were allowed *ad libitum* access to commercial rat chow and water.

***Quantification of transgene copy number.*** Copy number quantification per cell was done via genomic quantitative PCR (qPCR) as previously described [3] using the following primers (gene sequence 5'- to 3'-):

*ApoB_fwd* ATCTCAGCACGTGGGCTC

*ApoB_rev* TCACCAGTCATTTCTGCCTTTG

*Stop-ORF_fwd* ACGGCGAGTTCATCTACAAGG

*Stop-ORF_rev* GACCTCAGCGTCGTAGTGG

***Quantification of RNA concentrations by qRT-PCR.*** Tissue from hippocampal areas was collected from 120 μm thick frozen brain slices by manual microdissection using needles. Total RNA was isolated using TRIzol Reagent (Thermo Scientific, Germany) according to the manufacturer’s protocol. Quantification of respective mRNAs was conducted as described [4] with slight modifications. Briefly, 1 μg of total RNA was used for reverse transcription with SuperScript III and oligo(dT)20 primer (Invitrogen, Germany) according to manufacturer’s recommendations. Resulting undiluted cDNA solutions were subjected to real-time PCR analysis as duplicates (**Fig. 1F, S1J**) or triplicates (**Fig. S1D-F**).

For the quantification of *Stau1* and *Stau2* mRNA as well as the mRNA of housekeeping control gene *Ppia* (pair of primers #1), real time PCR was performed in a total volume of 20 μl using the Taqman Universal PCR master mix (Applied Biosystems, Germany) according to the manufacturer’s protocol with the primers indicated below and the following probes: #60 (for *Stau1*), #58 (for *Stau2*) and #42 (for *Ppia*) from universal probe library (Roche Applied Science, Germany). For the quantification of *EGFP* mRNA and the housekeeping control gene *Ppia* mRNA (pair of primers #1), quantitative real time PCR (qRT-PCR) was performed in a total volume of 20 μl using the Taqman Power SYBR-Green PCR master mix (Applied Biosystems, Germany) according to the manufacturer’s protocol with primers indicated below. Final concentrations of primer were 300 nM. For the quantification of *Arntl*, *Calm3* (total and intron-containing isoforms*), RhoA*, *Cplx1*, *Ppp2r1b*, *Rgs4* and *Stau2* mRNA and the housekeeping control gene *Ppia* mRNA (pair of primers #2) in Stau2 silenced and CAG control rats (as in **Fig. 1F, S1J**), qRT-PCR was performed in a total volume of 15 μl using SYBR green mastermix [1], in a Light Cycler 96 (Roche). Optimized primers are indicated below.

Detection of processed miRNA was performed using a Custom Taqman Small RNA Assay (AssayID CS70K4Q, Applied Biosystems) according to the manufacturer’s instructions. Briefly, 10 ng of total RNA was dissolved in 5 μl of RNase-free water and then mixed with 7 μl of recommended reverse transcription master mix (containing Superscript III reverse transcriptase) and 3 μl of the 5x reverse transcription primer provided with the Taqman Small RNA Assay. Reactions were run in a thermocycler, programmed for 30 min at 16°C, 30 min at 42°C and 5 min at 80°C. Once cDNA was synthesized, qRT-PCR reactions were run on an Applied Biosystems 7900 HT fast real-time PCR system (Applied Biosystems, Germany) in accordance with the recommended protocol [1]. Each qRT-PCR reaction was performed in triplicates and consisted of 1 μl undiluted cDNA, 1 μl Custom Taqman Small RNA Assay and 10 μl Taqman Universal PCR Master Mix (Applied Biosystems) in a total volume of 20 μl.

***Primers for qRT-PCR*** (gene sequence 5'- to 3'-):

*Stau2_fwd* AGGATCAGCTCGACAAGACC

*Stau2_rev* GGAAATCCAGGCTTTGGAC

*Stau1_fwd* TTCCAGAGCCCAGGGATT

*Stau1_rev* GAGAGATACACACTCGTTCTTGTTG

*Gfp_fwd* ACCCAGTCCGCCCTGAGCAA

*Gfp_rev* GCGGCGGTCACGAACTCCAG

*Ppia #1_fwd* CTTCCCAAAGACCACATGCT

*Ppia #1_rev* TGCTGGACCAAACACAAATG

*Ppia #2_fwd* GTCAACCCCACCGTGTTCTT

*Ppia #2_rev* CTGCTGTCTTTGGAACTTTG

*RhoA_fwd* AAGGACCAGTTCCCAGAGGT

*RhoA_rev* TGTCCAGCTGTGTCCCATAA

*Cplx_ fwd* GGCAGGGCATACGAGATAAG

*Cplx1_rev CTGGTGGGATAGCCTTCTTG*

*Rgs4_fwd* AGTCCCAAGGCCAAGAAGAT

*Rgs4_rev* AACATGTTCCGGCTTGTCTC

*Ppp2r1b_fwd* CAGCTGGGTGTGGAGTTTTT

*Ppp2r1b_rev* CATGAGGTTGTTGGTTGCTG

*Calm3-ORF_fwd* ACAGCGAGGAGGAGATACGA

*Calm3-ORF_rev* CATAATTGACCTGGCCGTCT

*Calm3-intron_fwd* GGAGACGGCCAGGTCAATTATG

*Calm3-intron_rev* GTCACCCAAAAGAAGGGCAAAC

*Arntl_fwd* TTAGCCAATGTCCTGGAAGG

*Arntl_rev* CCTGGAACAGTGGGATGAGT

***Primary neuron cultures*.** Embryonic day 17 (E17) hippocampal neurons were isolated from embryos of timed pregnant rats (Charles River), maintained in culture and transfected with calcium phosphate method as previously reported [5]. Cells were co-transfected at 14 days *in vitro* with the pBi-GFP-mirStau2 (miR(Stau2) is coexpressed with EGFP using the tetracycline-inducible gene regulation system) and pUHT-61-1 (constitutive expression of tetracycline-dependent transactivator tTA) plasmids; protein expression was analyzed by immunostaining 3 days later. After staining, images were acquired with a Zeiss Axioplan microscope with F-view Soft Imaging System, X-Cite120 Fluorescence Illumination System, 63X/1.4 oil Plan-Apochromat objective and analySIS B imaging software (Olympus).

***Immunohistochemistry and immunofluorescence***

Immunohistochemistry using DAB staining was performed as described [6] with slight modifications. Briefly, floating sections were treated with 0.5 % H_2_O_2_ in PBS for 15 min to reduce endogenous peroxide activity. After 2 washes with PBS, sections were incubated in PBS including 1% BSA and 0.3% Triton X-100 (day 1 buffer) for 1 h to reduce non-specific binding of antibodies. Next, the solution was exchanged to day 1 buffer containing the primary antibody. After overnight incubation, floating sections were washed twice with day 2 buffer (PBS including 0.3% BSA and 0.1% Triton X-100) and subsequently treated for 1 h with day 2 buffer containing the secondary antibody. Following 2 washes in day 2 buffer, the immunodetection was amplified using the avidin-HRP conjugated system (VECTASTAIN ABC (1 drop of solution A and 1 drop of solution B diluted in 20 ml day 2 buffer) for 1 h. After final washing steps (two with day 2 buffer and one with PBS), sections were stained with a diaminobenzidine (DAB) solution, containing 20 mg DAB (Sigma-Aldrich, Germany) and 12.5 μl 30 % H_2_O_2_ dissolved in 50 ml Tris-HCl (pH 7.6). The staining reaction was stopped with 3 subsequent PBS washes. Stained sections were mounted on uncoated glass slides and dried. Eukitt mounting medium (Sigma-Aldrich, Germany) was used to adhere the coverslip to the slide. For immunofluorescence, see antibodies below.

***Western blots***. Dissected tissues were homogenized in cold RIPA buffer (150 mM NaCl, 1.0% NP-40, 0.5% sodium deoxycholate, 0.1% SDS, 50 mM Tris-HCl pH 8.0) and centrifuged at 13,400g for 20 min at 4°C. Equivalent amounts of protein were separated via 10% SDS-PAGE and subjected to immunoblotting. Membranes were blocked using 2% BSA in TBS/0.1% Tween-20 (TBST) for at least 30 min at room temperature. Primary and secondary antibodies were diluted in blocking solution. Membranes were incubated with the respective primary antibodies (see below) overnight at 4°C, and the IRDye800 labeled secondary antibodies for 1 hour protected from light. All washes were performed in PBS/0.1% Tween. Membranes were scanned with the infrared-based Odyssey Imaging System (Li-Cor, Germany), and quantified using the Image Studio software.

***Primary antibodies*:**

- Rabbit polyclonal anti-EGFP (Invitrogen; 1:1,000); IHC
- Chicken polyclonal anti-EGFP (Millipore, Germany; 1:1,000); IF
- Rabbit monoclonal anti-NeuN (Abcam # ab177487; 1:500); IF
- Rabbit anti-Stau2 [7] (1:800); neurons in culture, sections
- Mouse anti-Stau2 (Monoclonal facility, Max F. Perutz laboratories, Vienna, Austria; 1:1000); WB
- Mouse anti-β-tubulin III (Sigma; 1:2,000); WB
- Mouse anti-Synapsin1 (Synaptic Systems; 1:500); IF
- Sheep anti-Digoxigenin Alkaline Phosphatase-conjugated, Fab fragment (Roche; 1:2,000); ISH

***Secondary antibodies*:**

- Biotinylated goat anti-rabbit IgG (Vector Laboratories, Germany; 1:600); IHC
- Donkey anti-chicken IgG A488-conjugated (Invitrogen, Germany; 1:200); IF
- Donkey anti-rabbit IgG Cy5-conjugated (Jackson Immuno, Germany; 1:200); IF
- Donkey anti-mouse IgG A647-conjugated (Invitrogen, Germany; 1:500); IF
- Donkey anti-rabbit Cy3-conjugated (Invitrogen; 1:2,000); IF neurons in culture
- Donkey anti-mouse IRDye800CW-conjugated (Odyssey; 1:15,000); WB

***Analysis of dendritic spines.*** Golgi impregnations were performed using FD GolgiStain Kit (FD NeuroTechnologies; USA). In each case, only the left hemisphere was used for staining. Subsequent 120 µm thick slices were made using a Leica VT 1000S vibratome, mounted on gelatin-coated slides and coverslipped with Merckoglas (Merck, Germany). Apical and basal dendrites of the hippocampal CA1 area, as well as apical dendrites of the CA3 area, were analyzed. Quantitative three-dimensional analyses were performed using a combined hardware-software system (NeuroLucida, version 9.12, MBF Bioscience, USA), controlling the x-y-z axis of the microscope (Axioscop Imaging, Zeiss, Germany) and the microscope-mounted digital camera (AxioCam HRc; Zeiss, Germany). The three-dimensional reconstruction was done under high magnification using a 100x plan Apochromat objective (oil immersion, numerical aperture 1.46). Only spines located on secondary or tertiary dendritic trees were evaluated and only one segment per individual dendritic branch was chosen for the analysis. Spine densities and mean spine length were calculated from the reconstructed dendrites with the help of NeuroExplorer (version 9.12; MBF Bioscience, USA). Concerning the analysis of the CA1 area, for each group 5 different brains were investigated. In each case 30 individual dendrites were mapped per region and brain. In total, 150 dendrites in each group and region and 23,846 (apical CA1) and 24,930 (basal CA1) spines, respectively, were analyzed. The analysis of apical dendrites of the CA3 area was performed on 4 different brains per group. In each case, up to 10 dendrites per brain were reconstructed and 2,010 individual dendritic spines were analyzed. For statistical analysis, either independent two-sample t-test or two-way ANOVA followed by a Sidak's multiple comparisons test were applied using Prism 5 (GraphPad Inc, USA). p<0.05 was determined as the statistical significance. Data were expressed as mean ± SEM.

**Electrophysiological *in vivo* recordings**

***Surgical preparation.*** Animals were anesthetized with 0.8-1.5% isoflurane delivered from a calibrated Fluotec 5 vaporizer (Fluotec-Ohmeda, Tewksbury, MA, USA) at a flow rate of 1-2 L/min. Rats were implanted with three stimulating electrodes in the medial perforant pathway, at the dorsomedial part of the right angular bundle (6.8 mm posterior and 3 mm lateral to Bregma; depth from brain surface, 2 mm) and with four recording electrodes in the pyramidal CA3 (3.3 mm posterior and 3.2 mm lateral to Bregma; depth from brain surface, 3.2 mm) and CA1 (3.6 mm posterior and 2.5 mm lateral to Bregma; depth from brain surface, 2.3 mm) areas. Stimulating electrodes were made of 50 μm, Teflon-coated tungsten wire (tip bared for ≈ 0.3 mm), whilst recording electrodes were made of 25 μm, Teflon-coated tungsten wire (Advent Research Materials, Eynsham, England). In order to detect the possible contamination of electrocortical recordings by vibrissae muscles activity, bipolar electromyographic recording electrodes were implanted in the whisker-pad. These electrodes were made of 50 μm, Teflon-coated, annealed stainless steel wire (A-M Systems, Carlsborg, WA, USA) with their tips bared of the isolating cover for ≈ 0.5 mm. Finally, animals were implanted with a 0.1 mm bare silver wire as ground. All wires were soldered to three six-pin sockets (RS Amidata, Madrid, Spain), and the sockets were fixed to the skull with the help of three small screws and dental cement [8, 9].

To verify the location of stimulating and recording electrodes once electrophysiological experiments were finished, brains of perfused rats were postfixed overnight at 4ºC and cryoprotected in 30 % sucrose in PBS. Respective sections (50 μm), obtained using a microtome (Leica, Wetzlar, Germany) were mounted on gelatinized glass slides and stained using a standard Nissl protocol with 0.1 % toluidine blue.

***Recording and stimulation procedures***. Field post-synaptic potentials (fEPSPs) were recorded with Grass P511 differential amplifiers through a high-impedance probe (2 × 10^12^ Ω, 10 pF). Electrical stimulus presented to the perforant pathway consisted of 100 μs, square, biphasic pulses presented alone, paired, or in trains. In each animal, we selected the two stimulating electrodes inducing better defined fEPSPs in the recording sites.

Stimulus intensities ranged from 0.02 to 0.4 mA for the construction of the input/output curves. For paired pulse facilitation, the stimulus intensity was set at 1/3 of the threshold for evoking a population spike, *i.e*. about 35 % of the intensity necessary for evoking a maximum fEPSP response [10]. Paired pulses were presented at six different inter-pulse intervals (10, 20, 40, 100, 200, and 500 ms).

For long-term potentiation (LTP) induction, the stimulus intensity was also set at 35% of the asymptotic fEPSP value. An additional criterion for selecting stimulus intensity for LTP induction was that a second stimulus, presented 40 ms after a conditioning pulse, evoked a larger (> 120 %) synaptic field potential than the first [11]. After 15 min of baseline records (1 stimulus/20 s), each animal was presented with a high-frequency stimulation (HFS) protocol consisting of five trains (200 Hz, 100 ms) of pulses at a rate of 1/s [9]. This protocol was presented 6 times in total, at intervals of 1 min. Thus, a total of 600 pulses were presented during a complete HFS session. In order to avoid evoking large population spikes and/or the appearance of electroencephalographic (EEG) seizures, the stimulus intensity during HFS was adjusted to the one used for generating baseline recordings. The evolution of fEPSPs after the HFS protocol was followed for 30 min at the same stimulation rate (1 stimulus/20 s). Additional recording sessions (15 min) were carried out for three additional days. For evoking long-term depression (LTD) we used a low-frequency stimulation (LFS) protocol consisting of a train of 900 pulses at 1 Hz, lasting for 15 min [12]. Recording sessions were organized as above described for LTP. Further details can also be found elsewhere [9, 13].

***Data collection and analysis.*** Electrocorticographic activity, fEPSPs, and 1 volt rectangular pulses corresponding to lever presses, pellet delivery, and brain stimulation were stored digitally on a computer through an analog/digital converter (CED 1401 Plus, Cambridge, England). Data were analyzed off-line with the Spike 2 (CED) program for quantification of EEGs, fEPSPs and performance in the Skinner box of each individual animal. The slope of evoked fEPSPs was computed as the first derivative (volts/s) of fEPSP recordings (volts). Three to five successive fEPSPs were averaged, and the mean value of the slope during the rise-time period (i.e., the period of the slope between the initial 10 % and the final 10 % of the fEPSP) was determined.

**Behavioral tests**

***Open field.*** Basal locomotor activity was assessed in an open field apparatus with four equal arenas (51 x 51 x 50 cm). Movements of the animals (one animal per arena) were digitally recorded for 30 minutes at a light intensity of 50 lx. The test was started by placing the rats in the center of the respective arena. Locomotor activity was analyzed by the video tracking software Viewer^2^ (Biobserve GmbH, Germany).

***Water maze***. Animals were trained in a circular water maze (1.5 m diameter, 50 cm in height; 25°C water temperature). The apparatus was located in a room containing extra maze visual cues consisting of various geometric shapes that were placed on the wall surrounding the maze. During training, an invisible escape platform (14 x 11 cm) was located in the water in a particular position 1 cm below water level. Swimming behaviors were recorded using the video tracking software Noldus Ethovision^®^ v.3. The water maze behavioral procedures consisted of training rats to find the escape platform for a total of three days. Each training session consisted of 6 swim trials, during which rats were placed in a starting pointing (changed randomly during each trials), and were allowed to swim to the escape platform, which was consistently located in the same position. Rats remained on the platform for 10 s before being returned to their home cage and being replaced in the maze after an inter-trial interval of 30 s. Rats that failed to find the escape platform within 60 s were manually guided to it. On the 4^th^ day, a probe trial was performed. Rats were placed into the maze that was lacking the escape platform and swimming behavior recorded by the video tracking software.

***Delay fear conditioning.*** Auditory fear conditioning and extinction were performed in respective chambers (Coulbourn Instruments, Allentown, PA) located in sound-attenuating cubicles (Med Associates, Burlington, VT) throughout all phases of the experiment. The floor of the chambers consisted of stainless steel bars able to deliver scrambled electric footshocks. Between experiments, shock grids and floor trays were cleaned with soap and water, and chamber walls were cleaned with wet paper towels. Footshocks were delivered from a precision animal shocker (H13-15, Coulbourn Instruments). Sound stimuli were generated with a conventional PC sound card, amplified by a HiFi amplifier (PR530A, Pyramid) and delivered via a loud speaker located in one of the walls of the conditioning chamber. Different conditioning chambers (context A and B) were used to allow separate assessment of contextual and cue-dependent fear memory. For Context A, a 17 cm × 18 cm × 32 cm sized conditioning chamber (H10-11M-TC, Coulbourn Instruments) with two transparent walls and a stainless-steel grid floor was applied. To achieve a different contextual perception for Context B, the described chamber was modified by inserting dark plastic panels to cover one of the transparent walls and the metal grid floor. On the other transparent wall, stripes of different shapes were installed. Respective chambers were also cleaned differently after being used. Context A was always cleaned with 1 % acetic acid, context B with 10 % ethanol solution. Conditioning protocols were delivered by a personal computer running FreezeFrame software (Actimetrics Software) through an IMAQ-A6822 interface card (National Instruments).

On day 1, rats were presented to five auditory conditioning stimuli (30 s, 4 kHz sinus tone, 77 dB) that each co-terminated with an electric footshock (1 s, 0.5 mA) in context A with an inter-trial interval of 3 min. On day 2 rats were first tested for contextual fear memory by placing the animals into context A for 5min without any tone presentation. After all experimental animals were tested for contextual fear memory cue-dependent fear memory was assessed. Here, animals were placed into context B for 2 min, in which the conditioning tone was presented twice for 30 s with an inter-trial interval of 30 s. For contextual extinction training, rats were returned into context A on day 3 for 30 min in absence of any footshock or tone signals.

In all sessions, movements of the tested animal were recorded with a digital video camera mounted at the ceiling of the cubicle and analyzed using FreezeView software (Actimetrics Software). In all experiments, the threshold for freezing behavior was set to 3.

***Trace fear conditioning.*** The basic experimental setup (e.g. conditioning chambers, hardware and software for recording and analysis) was the same as for delay fear conditioning, but there were minor differences in the procedure. On day 1 (acquisition phase) animals received 5 pairings of a tone CS (30 s, 5 KHz, 77 dB, ITI: 60-210 s) and a footshock US (1 s, 700 μA) in context A; CS and US were separated by a stimulus-free 20 s interval. On day 2 (context recall), rats were re-exposed to context A for 6 min without any cue presentations. On day 3 the rats’ cue-related freezing response was assessed by placing them in the alternative context B; following 120 s of baseline measurements, the CS was presented.

***Inhibitory avoidance task.*** The inhibitory avoidance paradigm was adapted from Moncada and colleagues [14]. Briefly, in the training session, individual rats were placed on a platform (45 cm long, 10 cm wide and 5 cm high) located attached the left wall within a modified fear conditioning chamber (45 cm long, 45 cm wide and 30 cm high) (TSE, Germany) designated as “Context A” (**Figure S4E**). Once the animal touched the shock grid with all its 4 paws, it received a scrambled footshock (0.5 mA; 3 s) and was subsequently removed from the box. 24 h later, the animal was placed again on the platform located in “Context A”. Once the animals step down the platform, the latency was recorded by a stop watch and the rat removed from the box without a shock delivery. Once all experimental animals underwent a testing in “Context A”, they were subsequently tested in the same way within the chamber modified to “Context B” (**Figure S4F**). In “Context B” the platform was attached to the front wall of the fear conditioning chamber. Additionally, environmental cues were modified.

***Novel object recognition and novel object location.*** Object recognition testing was performed in an open field apparatus with four equal arenas (51 x 51 x 50 cm) at a light intensity of 50 lx. The rats were habituated to the open field for 20 min on the day before testing. All stimulus objects used in the test were made out of ceramic or glass. The test consisted of a sample phase (P1; 3 min) and a discrimination phase (P2; 3 min) separated by an inter-trial interval of 15 min. During P1, rats were placed in the center of the open field arena and exposed to two identical objects (A1 and A2). After cessation of P1, rats were returned to the home cage for 15 min and objects was were removed. Then, rats were placed back in the open field arena for object discrimination phase P2. In the case of the novel object recognition task, the animal was exposed to a familiar object (A′, an identical copy of the object presented in P1) and a novel test object (B). In case of the novel object location task, one familiar object A was placed at the same position as in Phase P1 and another object A was placed to a different location within the open field arena.

Exploration of the objects (sniffing, licking) was recorded during P1 and P2. Sitting beside or standing on top of the objects was not scored as object investigation. Objects and arenas were cleaned with 70 % alcohol after each phase. Animals were videotaped during P1 and P2 and videos were analyzed by an observer blind to the genotype. Object preference was calculated by dividing the exploration time the animal spent with the novel/relocated object through was the total exploration time of both objects during the respective trial. The object preference indicated for the sample phase (sampling) represents the percentage of total exploration time spent on the object to be exchanged or relocated to control for a potential side preference.

***Delayed matching to place (DMTP) working memory-task in the morris water maze.*** The delayed matching to place working memory task in the water maze, originally developed in the laboratory of Richard Morris [15] was conducted according to Pezze and Bast [16] with slight modifications. Briefly, rats performed 4 trials each day, in which they had to search for an escape platform (11 x 14 cm) within a circular water maze (1.5 m diameter, 50 cm in height; 25˚C water temperature) located in a room with multiple extramaze cues to help the animal with spatial navigation. The platform changed every day to a position never encountered before by the animal but remained constant within each particular day (**Figure 3C**). The starting position for every trial was randomized to discourage an egocentric search strategy. In all trials the animal was given 120s to find the hidden platform. In case an animal was unable to locate the platform, it was manually guided towards it. During each trial the animal’s swimming behaviors were recorded using the video tracking software Noldus Ethovision^®^ v.3. The analysis focused particularly on trial 2 (T2) of each day, indicating the ability of the animal to encode spatial memory within a single trial. Trial 3 (T3) and 4 (T4) were performed only to reinforce the win-stay strategy of the paradigm.

Initially rats were trained in this task for 8 days with a constant inter trial interval (ITI) of 1 min between all trial (Phase 1; **Figure 3B**). During this training period, animals reached stable performance in the task. Next, the ITI between T1 and T2 was extended to 30 min for 3 days (Phase 2; **Figure 3B**). On the first day, time savings between T1 and T2 (expressed in percentage of escape latency of T1) was determined (**Figure 3C**). On the 3^rd^ day a probe trial was performed only on T2. Here, the escape platform was not present within the maze. The animal’s swimming behavior was recorded for 60 s before it was removed from the maze by a large kitchen strainer. Subsequently, T3 and T4 followed with the platform on the designated location learned in T1. As a measure for the correct memory of the platform position, the latency to enter the platform zone was recorded, covering a square area with an edge length of 30 cm centered on the middle of the platform position (**Figure 3D**). For the next 3 days, the ITI between T1 and T2 was extended to 6 h (Phase 3; **Figure 3B**). Analogous to Phase 2, the time savings in T2 compared to T1 were determined on the 1^st^ day and the probe trial performed on T2 of the 3^rd^ day. Finally, the ITI was set back to 1 min (Phase 1; **Figure 3B**). Now, time savings in T2 compared to T1 were determined on the 1^st^ day and the probe trial performed on T2 of the 2^nd^ day.

***Delayed nonmatching to place (DNMP) working memory-task on an 8-arm radial maze.*** The eight-arm radial maze used in this study consists of an octagonal center platform (diameter: 36 cm) with eight arms (length: 51 cm; width: 15 cm) emerging from it. The center platform is surrounded by walls and guillotine doors, located at the entrances of each arm that can be controlled through a wire system by an experimenter from a hidden place without interfering with the animals on the maze. Each arm is surrounded with a border (height: 2 cm) to prevent animals from falling of the maze. Also, each arm contains a food well (diameter: 5.3 cm; depth: 4 cm) located 3.2 cm from its distal end. All components of the maze are made of gray Plexiglas.

The maze was positioned in the middle of an experimental room enriched with various distal cues in a way that the center platform and the arms were 40 cm above floor level. In addition, a black cylinder (diameter: 36 cm; height: 40 cm) was located above the center platform, which could be lowered by the experimenter through the wire system.

Before behavioral testing, rats were kept on a food restriction diet to maintain 85% of the ad libitum body weights and were habituated to the apparatus for 2 consecutive days. During habituation, animals were allowed to freely explore the maze (with all guillotine doors open) until they collected all the food rewards (sweetened condensed milk termed “Milchmädchen”, Nestlé®) located in food well of each arm.

Next, animals were trained on a delayed non-matching to place (DNMP) task on an 8-arm radial maze. Here, animals learned to distinguish an unvisited from a previously visited arm of the radial maze in order to be rewarded. This was performed as follows: The guillotine door of one arm (sample arm) was opened, which allowed the rat to enter and collect the reward. Once the rat returned to the central platform of the maze, the black cylinder was lowered, covering the view of the animal on the arms. After a respective delay time, the black cylinder was lifted. Now the doors of the previously visited arm and an adjacent arm were open (study arms). Here, the animal was only rewarded it entered the arm not visited previously.

Each daily session was composed of a total of 4 trials, starting with a delay time of 1 min. Once rats reached a criterion of around 85 % of correct choice over 6 consecutive days, the delay time was increased to 5 min for 3 days of test and subsequently to 10 min for another 3 days. Experimental parameters scored are correct choices and latency to enter a study arm

***Operant conditioning*.** Training and testing took place in basic Skinner box modules measuring 29.2 × 24.1 × 21 cm (MED Associates, St. Albans, VT, USA). The operant chambers were housed within a sound-attenuating chamber (90 × 55 × 60 cm), which was constantly illuminated (19 W lamp) and exposed to a 45 dB white noise (Cibertec S.A., Spain). Each Skinner box was equipped with a food dispenser from which pellets (Noyes formula P; 45 mg; Sandown Scientific, Hampton, England) could be delivered by pressing a lever. Before training, rats were handled daily for a minimum of 7 days and food-deprived to 80–85% of their ad libitum body weight. Once the desired body weight was reached, animals were trained in the Skinner box once per day for 20 min on a fixed-ratio 1 (FR1) schedule, in which each lever press is rewarded by a pellet in the food tray. The start and end of each session was indicated by a tone (2 kHz, 200 ms, 70 dB) provided by the loudspeaker located in the recording chamber. FR1 training was continued until criterion (high and constant lever pressing activity for three consecutive days) was reached.

Next, animals were conditioned on the light/dark discrimination task for 10 additional days. In this protocol, only lever presses performed by the experimental animal during the lighted period (20 s) were reinforced with a pellet. Lever presses performed when the light was off were not reinforced and introduced a delay for the subsequent period ranging from 1 – 10 s.

After completion of training in the light/dark discrimination task, a dual signal recognition task was applied to the animals. For this paradigm, the Skinner box was provided with a Plexiglas wall separating the lever from the feeder tray. Walls lining the lever corridor were provided with two photoelectric beams located nearby (4 cm) the lever (1; **Figure 4G**) and at the beginning of the corridor (2; **Figure 4G**). In the dual signal recognition paradigm, the lever was removed from the Skinner box every second lighted period, before the animal crossed the photoelectric beam 2. Within respective sessions, animals were connected to the stimulating and recording system in order to record changes in fEPSP slopes at the moment they crossed the photoelectric beam 1. Data collected are from up to three sessions (each 20 min) per animal.

Conditioning programs, lever presses, and delivered reinforcements were controlled and recorded by a computer, using a MED-PC program (MED Associates, St. Albans, VT, USA). All operant sessions including were recorded with a synchronized video capture system (Sony HDR-SR12E, Tokyo, Japan) for off-line analysis.

**Supplemental references**

1. Sharangdhar T, Sugimoto Y, Heraud-Farlow J, Fernandez-Moya SM, Ehses J, Ruiz de Los Mozos I, Ule J, Kiebler MA: **A retained intron in the 3'-UTR of Calm3 mRNA mediates its Staufen2- and activity-dependent localization to neuronal dendrites.** *EMBO Rep* 2017, **18:**1762-1774.

2. Schönig K, Weber T, Frommig A, Wendler L, Pesold B, Djandji D, Bujard H, Bartsch D: **Conditional gene expression systems in the transgenic rat brain.** *BMC Biol* 2012, **10:**77.

3. Weber T, Schönig K, Tews B, Bartsch D: **Inducible gene manipulations in brain serotonergic neurons of transgenic rats.** *PLoS One* 2011, **6:**e28283.

4. Berger SM, Pesold B, Reber S, Schönig K, Berger AJ, Weidenfeld I, Miao J, Berger MR, Gruss OJ, Bartsch D: **Quantitative analysis of conditional gene inactivation using rationally designed, tetracycline-controlled miRNAs.** *Nucleic Acids Res* 2010, **38:**e168.

5. Heraud-Farlow JE, Sharangdhar T, Li X, Pfeifer P, Tauber S, Orozco D, Hormann A, Thomas S, Bakosova A, Farlow AR, et al: **Staufen2 regulates neuronal target RNAs.** *Cell Rep* 2013, **5:**1511-1518.

6. Spergel DJ, Kruth U, Hanley DF, Sprengel R, Seeburg PH: **GABA- and glutamate-activated channels in green fluorescent protein-tagged gonadotropin-releasing hormone neurons in transgenic mice.** *J Neurosci* 1999, **19:**2037-2050.

7. Fritzsche R, Karra D, Bennett KL, Ang FY, Heraud-Farlow JE, Tolino M, Doyle M, Bauer KE, Thomas S, Planyavsky M, et al: **Interactome of two diverse RNA granules links mRNA localization to translational repression in neurons.** *Cell Rep* 2013, **5:**1749-1762.

8. Fernandez-Lamo I, Montero-Pedrazuela A, Delgado-Garcia JM, Guadano-Ferraz A, Gruart A: **Effects of thyroid hormone replacement on associative learning and hippocampal synaptic plasticity in adult hypothyroid rats.** *Eur J Neurosci* 2009, **30:**679-692.

9. Gruart A, Munoz MD, Delgado-Garcia JM: **Involvement of the CA3-CA1 synapse in the acquisition of associative learning in behaving mice.** *J Neurosci* 2006, **26:**1077-1087.

10. Gureviciene I, Ikonen S, Gurevicius K, Sarkaki A, van Groen T, Pussinen R, Ylinen A, Tanila H: **Normal induction but accelerated decay of LTP in APP + PS1 transgenic mice.** *Neurobiol Dis* 2004, **15:**188-195.

11. Bliss TV, Gardner-Medwin AR: **Long-lasting potentiation of synaptic transmission in the dentate area of the unanaestetized rabbit following stimulation of the perforant path.** *J Physiol* 1973, **232:**357-374.

12. Manahan-Vaughan D: **Group 1 and 2 metabotropic glutamate receptors play differential roles in hippocampal long-term depression and long-term potentiation in freely moving rats.** *J Neurosci* 1997, **17:**3303-3311.

13. Madronal N, Delgado-Garcia JM, Fernandez-Guizan A, Chatterjee J, Kohn M, Mattucci C, Jain A, Tsetsenis T, Illarionova A, Grinevich V, et al: **Rapid erasure of hippocampal memory following inhibition of dentate gyrus granule cells.** *Nat Commun* 2016, **7:**10923.

14. Moncada D, Viola H: **Induction of long-term memory by exposure to novelty requires protein synthesis: evidence for a behavioral tagging.** *J Neurosci* 2007, **27:**7476-7481.

15. Steele RJ, Morris RG: **Delay-dependent impairment of a matching-to-place task with chronic and intrahippocampal infusion of the NMDA-antagonist D-AP5.** *Hippocampus* 1999, **9:**118-136.

16. Pezze M, Bast T: **Dopaminergic modulation of hippocampus-dependent learning: blockade of hippocampal D1-class receptors during learning impairs 1-trial place memory at a 30-min retention delay.** *Neuropharmacology* 2012, **63:**710-718.
